# High Sodium Ion Storage by Multifunctional Covalent Organic Frameworks for Sustainable Sodium Batteries

**DOI:** 10.1021/acsami.3c17710

**Published:** 2024-03-18

**Authors:** Mohammad K. Shehab, Hani M. El-Kaderi

**Affiliations:** Department of Chemistry, Virginia Commonwealth University, Richmond, Virginia 23284, United States

**Keywords:** covalent organic frameworks, sodium-ion batteries, redox-active covalent organic frameworks, electrochemical energy storage, organic electrodes

## Abstract

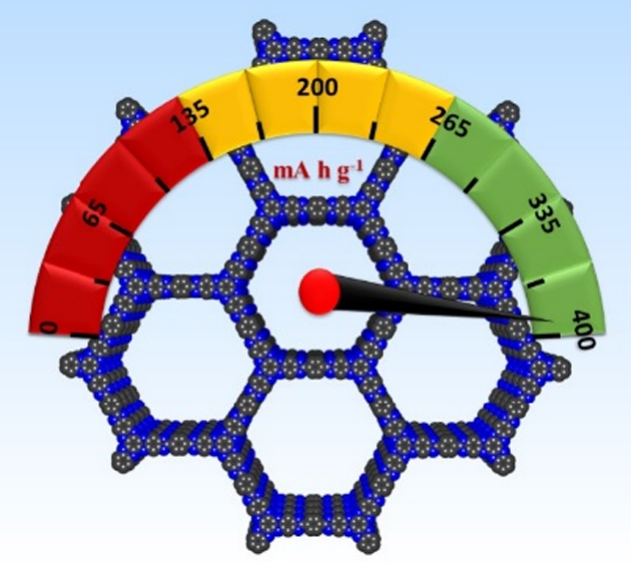

Rechargeable sodium batteries hold great promise for circumventing the increasing demand for lithium-ion batteries (LIBs) and the limited supply of lithium. However, efficient sodium ion storage remains a great impediment in this field. In this study, we report the designed synthesis of a multifunctional two-dimensional covalent organic framework featuring hexaazatrinaphthalene cores linked by imidazole moieties and demonstrate its effective performance in sodium ion storage. Benzimidazole-linked covalent organic framework (BCOF-1) was synthesized by a condensation reaction between hexaazatrinaphthalenehexamine (HATNHA) and terephthalaldehyde (TA) and exhibited a high theoretical specific capacity of 392 mA h g^–1^. BCOF-1 crystallizes, forming eclipsed AA stacking and mesoporous hexagonal one-dimensional channels with high surface area (840 m^2^ g^–1^), facilitating fast ionic mobility and charge transfer and enabling high-rate capability at high current rates. BCOF-1 exhibits pseudocapacitive-like behavior with a high specific capacity of 387 mA h g^–1^, an energy density of 302 W h kg^–1^ at 0.1 C, and a power density of 682 W kg^–1^ at 5 C. Our results demonstrate that redox-active COFs have the desired structural and electronic merits to advance the use of organic electrodes in sodium-ion storage toward sustainable and efficient batteries.

## Introduction

Covalent organic frameworks (COFs) are crystalline organic polymers built by connecting organic building blocks together with strong covalent bonds.^1−3^ The structural and chemical composition of COFs can be tailored to target many applications such as electrochemical energy storage,^4−8^ drug delivery,^9,10^ gas separation and storage,^11,12^ water harvesting,^13,14^ heavy metal removal,^15,16^ and catalysis.^17−19^ For instance, COFs have shown great promise in supercapacitors,^20−22^ pseudocapacitors,^4,8,23^ and rechargeable batteries.^24−27^ The highly cross-linked nature of COFs prevents dissolution in electrolytes, while π-conjugation and porosity secure rapid charge transfer and ion transport. Because ion storage in COFs is not limited to intercalation, as in the case of graphite, COFs can be applied as electrode materials to store large ions like sodium, potassium, and aluminum.^28^ The readily accessible pores and abundant redox-active sites in COFs have been proven to be crucial for attaining high-energy and power-density batteries. As such, COFs are uniquely suited to advance new battery technologies beyond lithium-ion batteries (LIBs) to mitigate the increasing demand for sustainable batteries. LIBs are extensively used as a power supply for electric vehicles, portable electronics, and grid-scale energy storage systems because of their matured technology.^29−31^ However, the cost of lithium and its uneven geographical distribution makes it vital to consider more sustainable alternatives to meet market demands.^32,33^ Therefore, cost-effective battery technologies based on abundant metals like sodium,^4,8,34−37^ aluminum,^38^ potassium,^39,40^ and magnesium^41^ have received considerable attention recently. Among these options, rechargeable sodium-ion batteries (SIBs) have attracted attention due to their low cost, abundance of sodium, and competing performance with LIBs.^42^

In an effort to enhance SIBs' performance and minimize transition metals used in cathodes like cobalt, a variety of redox-active organic materials have been synthesized and investigated due to their advantageous merits, like natural abundance, structural tunability, lightweight, and environmental friendliness.^43−48^ Unfortunately, small organic molecules tend to dissolve in electrolytes, and adding additives such as conducive materials and binders is needed to retain the electrode’s structural stability and electrical conductivity. Such challenges have been addressed using organic polymers and COFs, with the latter being more advantageous as the reticular design of COFs permits inserting dense, accessible redox-active sites into crystalline porous π-conjugated frameworks, which enhances the ion diffusion and charge transfer and, thereby, affords superior electrodes. Despite the intense research activity in this area, extending the use of COFs to SIBs has been limited, and hence, the design of new COFs to improve the electrochemical performance of SIBs in terms of capacity and rate capability is very desirable.

Herein, we report the synthesis of a crystalline redox-active benzimidazole-linked COF (BCOF-1) by a condensation reaction between HATNHA and TA and demonstrate its effective use in SIBs. The rationale for choosing HATNHA and TA as building units is to increase the number of redox-active sites, since HATNHA already has six redox-active sites, and upon condensation with TA, the number of active sites increases due to imidazole ring formation. On the other hand, TA is a simple aldehyde that ensures low molar mass per repeating unit, both of which are necessary for optimizing specific capacity. BCOF-1 exhibits high specific capacity, rate capability, and cycling stability, competing with the best-performing organic electrodes in the field. As a result, this work provides fundamental insights into the structure–function relationship of COFs in electrochemical energy storage and delivers new materials for sustainable and efficient batteries beyond LIBs.

## Experimental Section

### Materials

The chemicals used were purchased from commercial suppliers and utilized without further purification. 1,2-Diaminobenzene (Alfa Aesar, 98%), benzenesulfonyl chloride (Alfa Aesar, 98%), pyridine (Sigma-Aldrich, 99+), palladium on activated carbon (Pd/C, Acros Organics, 10% Pd), hexaketocyclohexane octahydrate (HKH·8H_2_O, Fisher Scientific, 97%), terephthalaldehyde (ThermoFisher Scientific, 98%), anhydrous 1,4-dioxane (Acros Organics), mesitylene (Acros Organics), sulfuric acid (H_2_SO_4_, Fisher Scientific, 94–98%), diethylene glycol dimethyl ether (DEGDME, Sigma-Aldrich, anhydrous 99.5%), conductive carbon black (Ketjenblack-600JD), sodium hexafluorophosphate (NaPF_6_, Alfa Aesar, 99+%), and sodium alginate (Alfa Aesar, high viscosity).

### Synthesis of the Building Blocks

The building blocks were synthesized based on the literature with modifications (Supporting Information).

### Synthesis of BCOF-1

**Figure 1:**
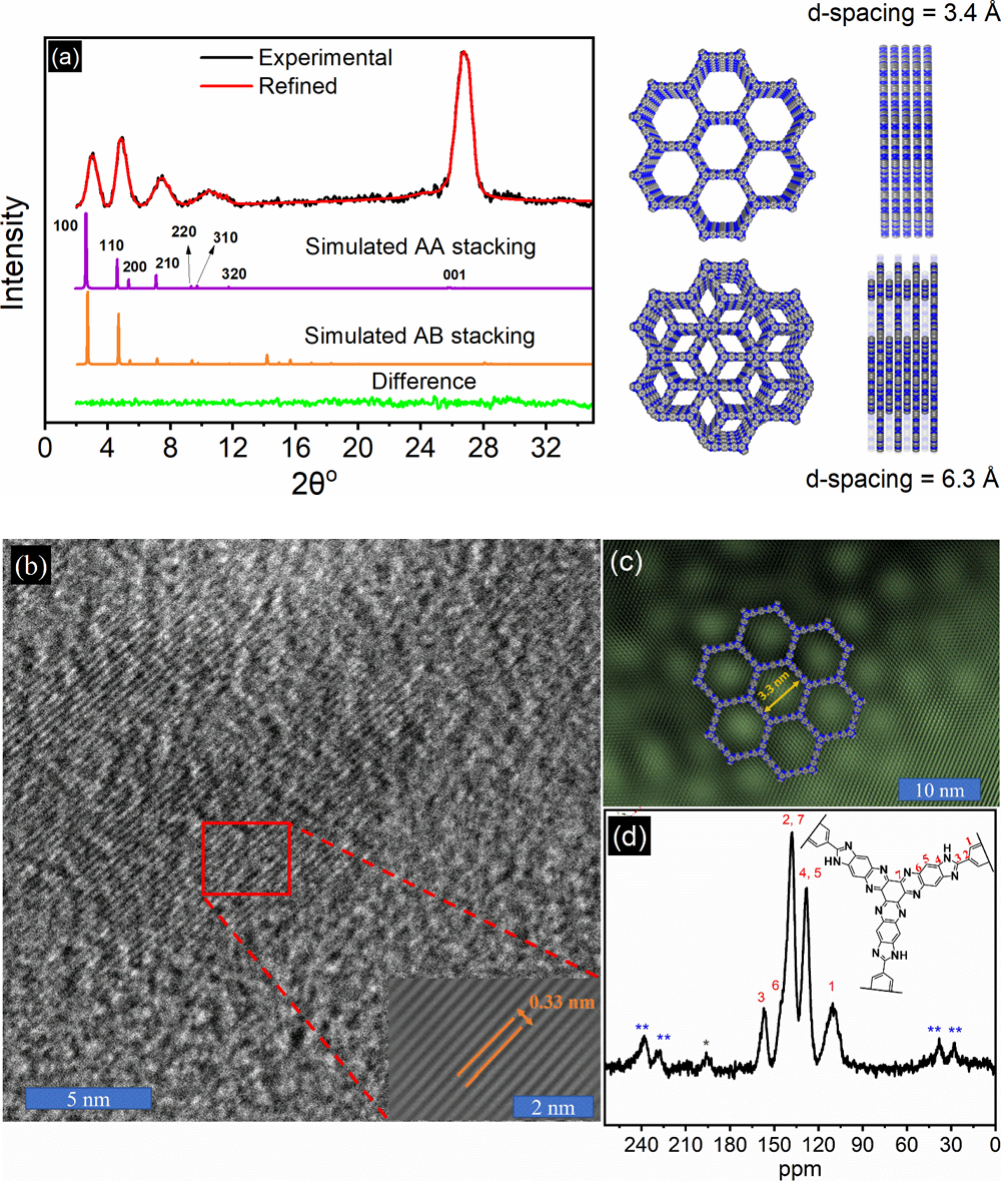
(a) PXRD patterns of BCOF-1, where the experimental is in black, and the Pawley refinement in red shows a very minimal difference (green line). The simulated PXRD patterns for the AA eclipsed and AB staggered structures are in purple and orange, respectively. (b) HRTEM images exhibit well-ordered lattice fringes, the inset represents the FFT image for the diffracted patterns, and (c) well-ordered 2D hexagonal channels with a diameter of 3.3 nm. (d) ^13^C SS-NMR for BCOF-1, the asterisk denotes the unterminated aldehyde in TA, and double asterisks indicate spinning sidebands.

A dried Pyrex tube was charged with HATNHA (25.0 mg, 0.0526 mmol) and terephthalaldehyde (10.5 mg, 0.0783 mmol, 1.5 equiv), and 1,4-dioxane (2.0 mL) and mesitylene (0.5 mL) were charged in sequence. The mixture was sonicated for 3 min, and the tube was flash-frozen at 77 K using a liquid N_2_ bath. After the first freezing, 0.5 mL of 3 M CH_3_COOH was added, followed by three freeze–pump–thaw cycles. Eventually, the tube was flame-sealed under a vacuum and placed in the oven at 120 °C for 5 days. A dark brown precipitate formed at the bottom of the tube, which was isolated by filtration and washed with DMF (10 mL × 2). The solid was soaked in DMF (20 mL) at room temperature for 2 days, during which the solvent was replaced twice daily. After 2 days, DMF was replaced with CH_2_Cl_2_ and kept exchanged with the same solvent 2 times per day for 2 days. Finally, the product was soaked in absolute ethanol and activated by using supercritical CO_2_ to afford a reddish-brown fluffy precipitate. ATR-IR; 3200 (−N–H), 1610 cm^–1^ (C=N imidazole ring), 1416 cm^–1^ (C–C stretching for the new benzene linker), 1461 and 1241 cm^–1^ for (C=C), 1241 cm^–1^ (C=N) in the pyrazine ring, respectively (SI, Figure S18). ^13^C SS-NMR 156, 144, 137, 128, 110 ppm (Figure 1d).

### Electrode Preparation and Coin Cell Assembly

BCOF-1 was utilized as a working electrode and tested as the positive electrode in a sodium metal half-cell. The positive electrode composite consisted of 50 wt % active material (BCOF-1), 30 wt % Ketjenblack-600JD, and 20 wt % binder (sodium alginate). A slurry was made of all aforementioned components along with double deionized water (DDW) and kept under stirring overnight at room temperature. Then, the slurry was cast on an aluminum foil current collector using a doctor blade and dried in a vacuum oven at 70 °C. The positive electrode was then cut into a circular disc with a diameter of 15 mm and assembled in a CR2032-type coin cell. Sodium metal (negative electrode) was cut into a disc too and used as a counter and reference electrode; 1.0 M NaPF_6_ in DEGDME solution served as an electrolyte to facilitate the sodium ions movement between the electrodes (15 μL/0.5 mg of the active material). A polypropylene separator was used as a membrane to separate between electrodes. The entire battery assembly process was done inside an argon-filled glovebox using an electric crimper (MTI Corp.)

### Physical and Spectral Characterization

The nuclear magnetic resonance data (^1^HNMR and ^13^CNMR) were obtained by NMR-400 MHz (Bruker), and ^13^CSS-NMR data were obtained by Bruker Avance HD 400 MHz w/4 mm HX and 1.6 mm HFX solid probes. Attenuated total reflection infrared spectroscopy (ATR-IR) was used to collect the infrared spectra. The surface topography images were captured at high magnifications by scanning electron microscopy (SEM, Hitachi SU-70 FE-SEM). A 3Flex surface analyzer (Micrometrics) was utilized at 77 K to collect the nitrogen adsorption/desorption isotherms and the Brunauer–Emmett–Teller (BET) surface area measurements. BCOF-1 was activated by degassing at 80 °C for 4 h and then was increased to 110 °C for 7 h at a rate of 5 min °C^–^^1^ under a 10^–6^ bar vacuum. X-ray photoelectron microscopy (XPS) measurements were carried out using a PHI VersaProbe III scanning XPS microprobe. For Raman spectroscopy studies, Thermo Scientific DXR Smart Raman was used at a 532 nm laser with a power of 5 mW to excite the samples.

### Electrochemical Measurements

All electrochemical studies of the assembled coin cells were carried out and examined at room temperature using an electrochemical workstation (CHI 600C). This includes cyclic voltammetry (CV), galvanostatic charge–discharge, rate capability, long-term cycling stability, and electrochemical impedance spectroscopy (EIS).

## Results and Discussion

While numerous amorphous benzimidazole-linked polymers (BILPs) have been reported in the literature, their crystallization has been a great challenge because of the difficulty associated with controlling the polymerization processes during imidazole ring formation.^49,50^ More recently, new synthetic strategies were developed to crystallize BILPs and enable complete characterization of their solid-state packing and porosity.^51,52^ These methods include condensation reactions of aryl aldehydes or carboxylic acids with aryl-diamine building blocks in polyphosphoric acid or by Debus–Radziszewski multicomponent reactions.^51,52^ The impact of crystallinity on the ion transport, like proton conductivity, indicated that crystalline BILPs exhibit a remarkable enhancement in proton conductivity as compared to the amorphous analogues due to the facilitated diffusion of protons along the 1D channels.^51^ Similarly, imidazole-functionalized linkers in imine-COFs showed high Li^+^ diffusion rates when used as solid-state electrolytes for LIBs.^53^ These studies infer that the solid-state ordering of COFs is vital for rapid ion diffusion and transport. Nevertheless, the structural and functional diversity of imidazole-linked COFs is still very scarce and has somewhat limited their applications. Therefore, it was of immense importance to develop a new synthetic strategy that integrates redox-active moieties into the skeleton of BILPs without compromising the porosity and crystallinity, which are central for energy storage.

**Scheme 1:**
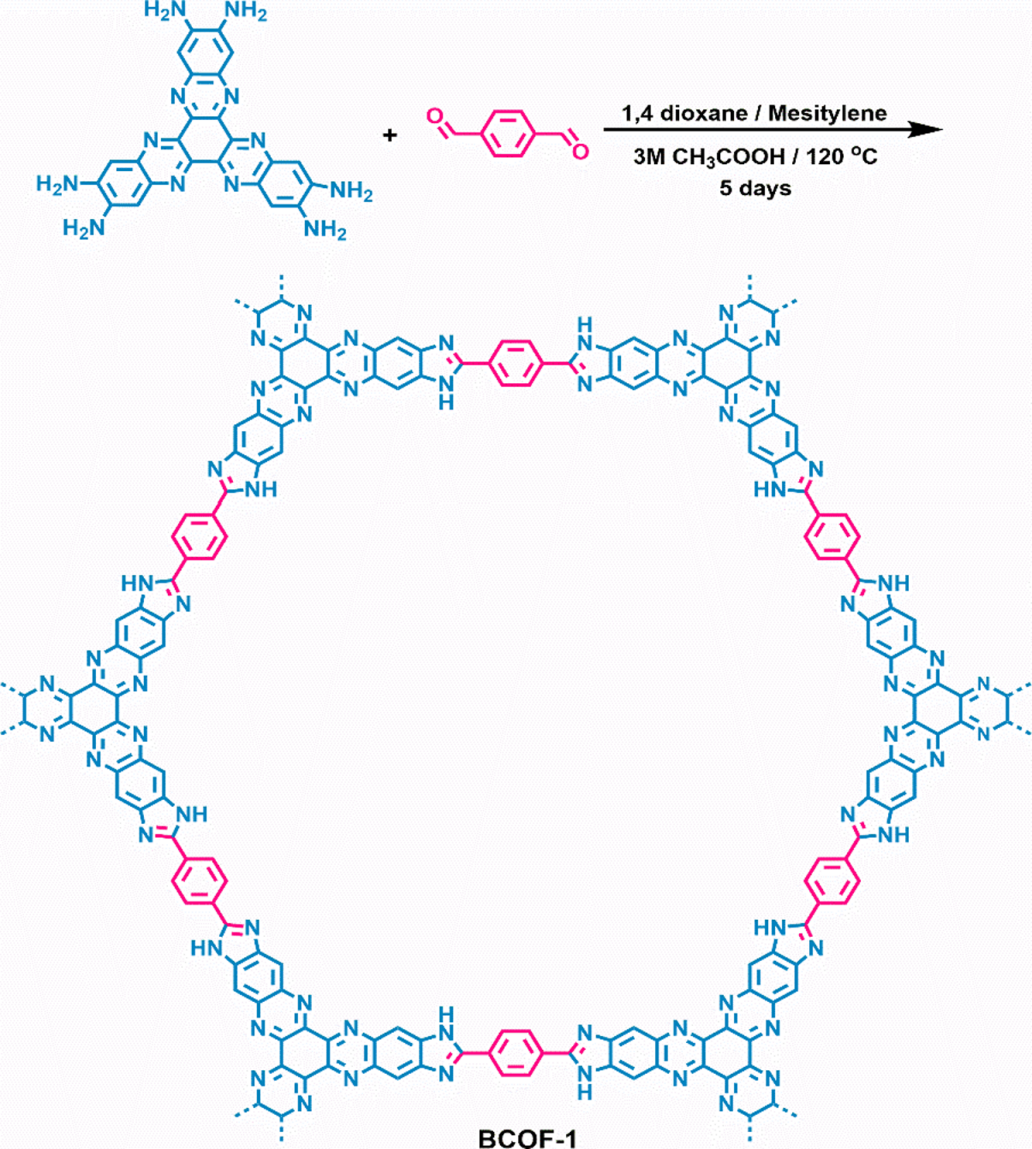
Synthesis of BCOF-1 by the Condensation Reaction between HATNHA and Terephthalaldehyde

In this study, the redox-active hexaazatrinaphthalene (HATN) moiety was incorporated into BCOF-1 using a polycondensation reaction between HATNHA^54^ (SI, Figures S1–S17) and TA, as shown in Scheme 1. BCOF-1 was characterized using a suite of spectral and analytical characterization techniques. The network connectivity between the building blocks was confirmed by carbon solid-state nuclear magnetic resonance (^13^C SS NMR) (Figure 1d) and attenuated total reflection infrared (ATR-IR) spectroscopy (SI, Figure S18). The SS-NMR and IR results for the precursors and BCOF-1 indicated the successful formation of the BCOF-1. The stretching bands of carbonyl groups in the TA and the amine groups in the HATNHA at 1650 and 3200 cm^–1^, respectively, disappeared upon the formation of BCOF-1. On the other hand, a broad band centralized at 3100 cm^–1^ and N–H wag for secondary amine at 850 cm^–1^ support the formation of the imidazole ring as part of the framework. The crystallinity of BCOF-1 was confirmed by powder X-ray diffraction (PXRD) and high-resolution transition emission microscopy (HRTEM), as shown in Figure 1. The potential formation of AA (eclipsed) and AB (staggard) stacking was simulated to determine the packing of 2D sheets. Simulation of AA stacking was done using *P*_6_/*mmm* space group with lattice parameters of *a* = *b*= 37.716, *c* = 3.442 Å and α = β = 90°, γ = 120°. On the other hand, the model of AB stacking was simulated using the *P*6_3_/*mmc* space group with a lattice parameter of *a* = *b*= 37.882 Å, *c* = 6.360 Å and α = β = 90°, γ = 120°. As can be found, the experimental PXRD pattern for BCOF-1 concurs well with the simulated pattern of the eclipsed model, showing high order along the *z*-axis (001). The experimental PXRD pattern agreed with the calculated pattern obtained for the eclipsed model showing reflections at 2θ = 2.71°, 4.69°, 5.41°, 7.16°, and 26.10°, which correspond to the (100), (110), (200), (210), and (001) crystal planes, respectively. Pawley refinement profile fitting factors *R*_p_ and *R*_wp_ were found to be 2.53 and 3.31%, showing a very small difference between the fitted and the experimental patterns (Figure 1a, SI, Table S1). The periodicity of the porous framework of BCOF-1 was also confirmed by high HRTEM, as depicted in Figure 1. HRTEM images show high-ordered lattice fringes with an interlayer distance of 3.30 Å between the (001) crystal planes (Figure 1b). Furthermore, a high-ordered honeycomb-like structure was seen with a uniform channel diameter of about 3.3 nm, which is in agreement with the pore size of the simulated AA stack model and PXRD results (Figure 1c). To investigate the porosity of BCOF-1, nitrogen adsorption/desorption isotherms were collected. The BET surface area was found to be (SA_BET_ = 840 m^2^ g^–1^). Furthermore, the pore size distribution showed a combination of both micropores and mesoporous (SI, Figure S19b), which causes the apparent hysteresis between adsorption and desorption at higher relative pressure in the isotherm^55,56^ (SI, Figure S19a). BCOF-1 exhibits nanorod-like morphology according to SEM imaging studies (SI, Figure S20).

**Figure 2:**
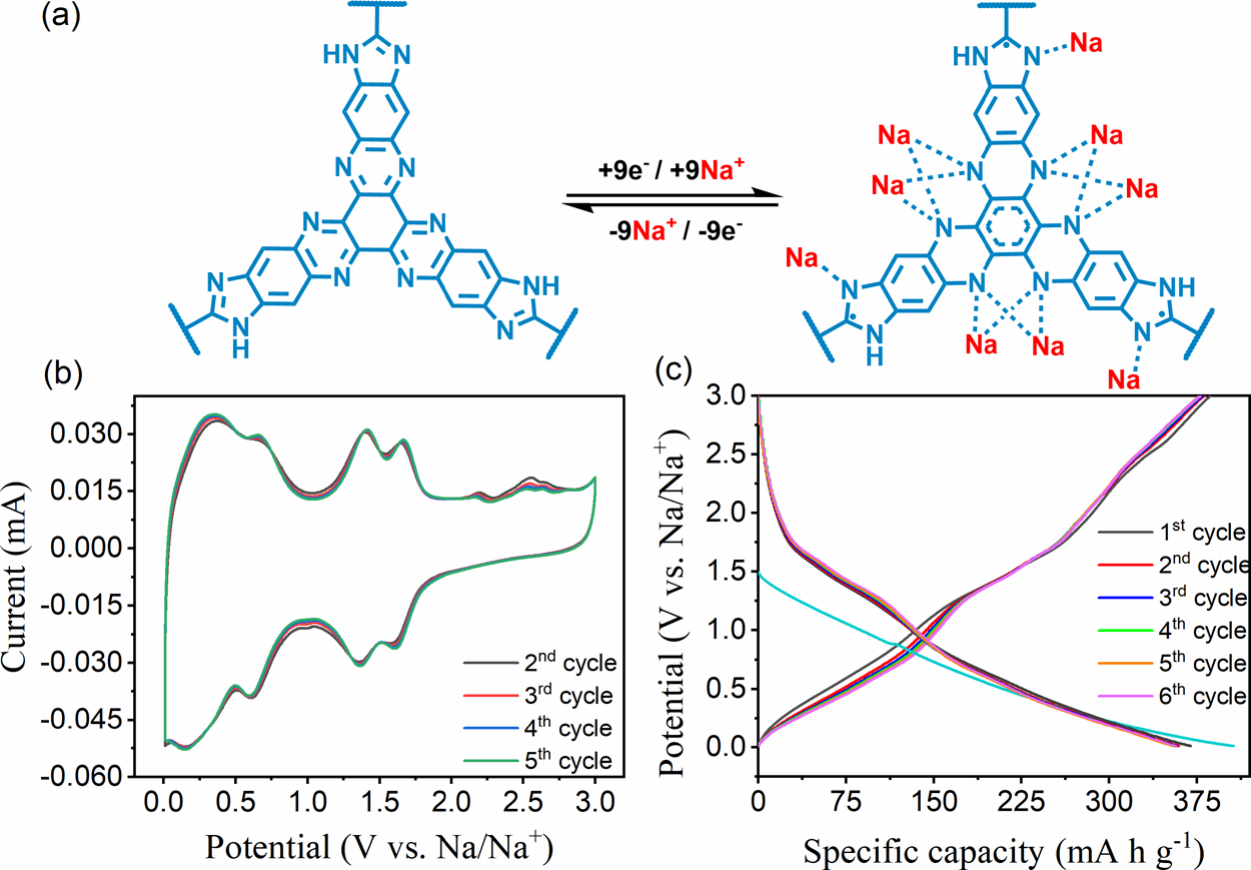
(a) Redox mechanism of the aza active sites. (b) Cyclic voltammograms at a scan rate of 0.1 mV s^–1^ of the potential range between 0.01 and 3.0 V. (c) Cycles of galvanostatic charge/discharge at the current rate of 0.1 C.

Once the textural properties of BCOF-1 were established, its performance in sodium ion storage was investigated by using coin cells and subjected to a series of electrochemical studies. Cyclic voltammetry (CV) studies of the assembled coin cell were used to investigate the reversibility and electron transfer kinetics of the redox reactions. CV peaks attest to the reversible redox reactions of the aza active sites (C=N) in the phenazine and imidazole units. Figure 2b shows three highly reversible redox curves at around 0.67–0.50/0.58–0.72, 1.51–1.24/1.30–1.54, and 1.64–1.51/1.54–1.71 V. The first reversible redox peak is attributed to the redox reaction on the aza bond of the imidazole ring. To ensure that the CV of the redox-active moiety, monomer in BCOF-1 was carried out at a scan rate of 0.1 mV s^–1^ and showed the same reversible peak of the framework at 0.67–0.50/0.58–0.72 (SI, Figure S21). In comparison, the other two peaks have been assigned to the HATN core in monomers, COFs, and porous polymers due to the insertion of two sets of three sodium ions consecutively.^36^ Furthermore, irreversible anodic peaks were initially observed at 2.6 V and then vanished by the fifth cycle, which could be ascribed to the formation of the solid electrolyte interface (SEI) (SI, Figure S22). The multifunctional design of BCOF-1 offers a very high theoretical specific capacity of 393 mA h g^–1^ based on nine redox-active sites (Figure 2a). To investigate the performance of BCOF-1 as electrode material in SIBs, galvanostatic charge/discharge studies (6 cycles) were carried out at a current rate of 0.1 C in a potential window of 0.01 to 3.0 V vs Na/Na^+^. In the first cycle, a charge specific capacity of 387 mA h g^–1^ and a discharge specific capacity of 370 mA h g^–1^ were obtained. The resultant charge/discharge capacities speak for 98.5 and 94.1%, respectively, of the theoretical capacity. On the other hand, starting from the second cycle onward, the capacities for all other cycles exhibited highly reversible charge/discharge capacities of about 377/360 mA h g^–1^ (Figure 2c). The observed hysteresis in the first cycle could be attributed to the electrolyte reduction on the electrode surface during the formation of the SEI.^57^ It is worth noting that the galvanostatic charge/discharge slopes exhibit three reversible curves that are consistent with the CV curves.

**Figure 3:**
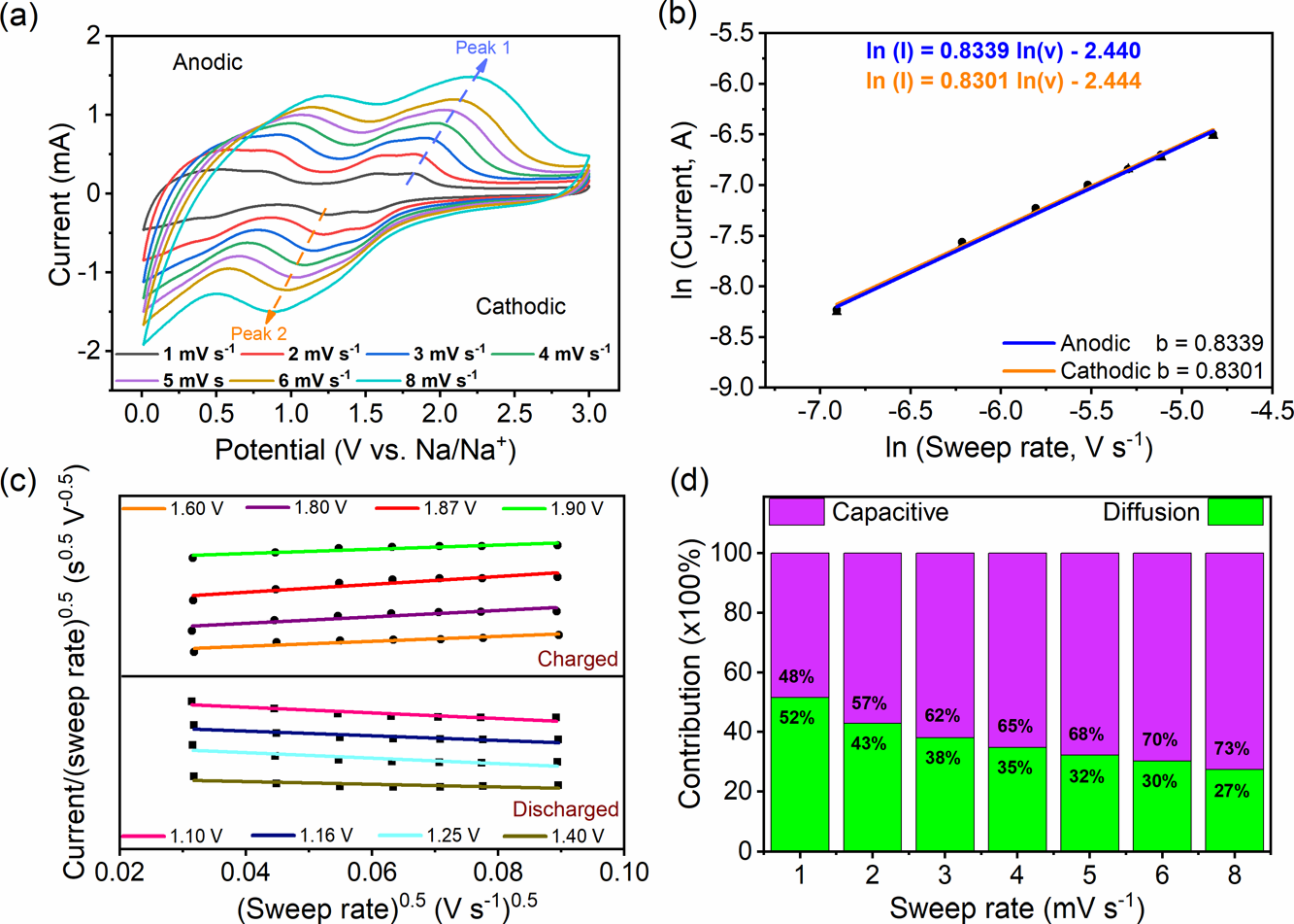
(a) CV curves at different sweep rates (1.0–8.0 mV s^–1^) of the BCOF-1 electrode in the DEGDME-based electrolyte. (b) Corresponding linear fit of the ln v vs ln i at 1, 2, 3, 4, 5, 6, and 8 mV s^–1^. (c) Plot of *i*(*V*)/ν^0.5^ vs ν^0.5^ of the BCOF-1-based electrode at different fixed potentials and (d) overall total charge storage contribution at different sweep rates.

The diffusion kinetics and the energy storage mechanism were obtained from CV measurements at different sweep rates (*v*) in the range of 1 to 8 mV s^–1^ (Figure 3a). In general, increasing the sweep rate magnifies the current peak (*i*) and broadens the cyclic voltammogram, as described in the equation below. However, the reversibility of the redox peaks still existed.
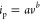


For the diffusion-controlled process, the peak current varies as *v*^0.5^ (*b* = 0.5), which is the battery-like behaving process. However, for the surface-limiting redox, the peak current varies directly as *v*^1^ (*b* = 1), which is a capacitive-like process.^58^ When *b* falls between 0.5 and 1, the pseudocapacitive behavior dominates due to the fast surface-controlled Faradaic process.^59^ Several cyclic voltammograms at different sweep rates were used to calculate the *b*-values at the current maxima in the anodic and cathodic systems. The *b* values were found to be 0.8339 for anodic and 0.8301 for cathodic systems, while the small difference in the *b* values indicates a highly reversible redox system. Moreover, the redox peak potential separation is near zero, which indicates a pseudocapacitive-like behavior (Figure 3b).^59^ These results were used to quantify the contribution from diffusion and capacitive processes to total energy storage using the following equation.
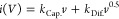
where *i*(*V*) is the current at a fixed potential, *k*_Cap._ and *k*_Dif._ are adjusted capacitive and diffusion parameters, *k*_Cap._*v* is the capacitive contribution, and *k*_Dif._*v*^0.5^ is the diffusion contribution. By rearranging the above equation, *k*_Cap._ (slope) and *k*_Dif._ (intercept) can be easily calculated. *k*_Cap._ and *k*_Dif._ at different fixed potential values were calculated for oxidation potentials (charge) of 1.60, 1.80, 1.87, and 1.90 V; reduction potentials (discharge) were calculated at 1.10, 1.16, 1.25, and 1.40 V (Figure 3c). As a result, it is possible to specify the current fraction arising from the sodium ion diffusion (Faradaic current) and the current fraction arising from the capacitive process (non-Faradaic current) at a certain potential and then utilize these to make the overall charge storage diagram (Figure 3d).

The contribution diagram of the BCOF-1 electrode at sweep rates of 1, 2, 3, 4, 5, 6, and 8 mV s^–1^ shows the Faradaic (green) and non-Faradaic (violet) current contribution (Figure 3d). At a lower sweep rate of 1 mV s^–1^, the Faradaic current produced due to sodium ion diffusion (52%) was almost dominant, comparable to the non-Faradaic current arising from the capacitive process (48%). However, increasing the sweep rates makes the capacitive current more dominant. As the sweep rate increases from 1 to 8 mV s^–1^, the capacitive contribution increases to 48, 57, 62, 65, 68, 70, and 73%, respectively. This is in agreement with the cyclic voltammogram at a higher sweep rate when the peaks become gradually broader, and their shape tends to be more rectangular, which is a distinct feature of the capacitance behavior.^58^ Therefore, the higher the sweep rate, the higher the capacitive contribution and, thus, more charge storage in the porous BCOF-1-based electrode.^60^ Electrochemical impedance spectroscopy (EIS) studies with a frequency range of 0.01 Hz to 1 MHz were carried out to further investigate the BCOF-1 electrode kinetics. Nyquist plots were made for the fresh and cycled (400 cycles) coin cells (SI, Figure S23a,b). The fresh coin cell plot exhibits small diameter semicircles, whereas the cycled coin cell plot shows a large semicircle diameter, which is attributed to a larger charge transfer resistance (*R*_ct_) built up upon cycling. The reason behind the wider *R*_ct_ is the partial decomposition of the electrolyte and the formation of inorganic and organic salts upon cycling, which might block the pores partially and minimize the sodium ion mobility toward the redox-active sites in and out.^61^ Furthermore, both coin cells possessed an approximately 45° inclined line from the imaginary impedance resistance axis (−*Z*″). The inclined line resembles a higher diffusive resistivity and, thus, a slower diffusion process of sodium ions into the bulk material of the electrode.^59,62^ As a result, the small semicircle for the fresh coin cell and the inclined line from the vertical axis confirm the pseudocapacitive behavior for a speedy surface-controlled Faradaic process. Resistance can arise due to three factors; the resistance of the electrolyte (*R*_el_),^21^ the charge transfer resistance (*R*_ct_), and Warburg resistance (*R*_w_) for the diffusion process. Randles equivalent circuits were used to simulate and numerically analyze the Nyquist plots (SI, Figure S23c,d). The *R*_el_ can be obtained by the first intersection point of the semicircle with the real impedance resistance axis (*Z*′). The *R*_el_ values for the fresh and cycled coin cells were 8.396 and 9.075 Ω, respectively. The *R*_ct_ can be determined by the diameter of the semicircle; a smaller diameter indicates a faster charge transport. The fresh coin cell exhibits a lower diffusion resistance (8.078 Ω), unlike the cycled coin cell over 400 cycles, which showed a significant diffusion impedance (422.3 Ω). The *R*_w_ values were found to be very small, 0.0743 and 0.00882 Ω for fresh and cycled coin cells, respectively, due to *R*_w_ is always associated with an *R*_CT_ and the diffusion process.^61^ It is worth mentioning that the little deviation of Warburg diffusion from 45° confirms that the BCOF-1-based electrode does not exhibit ideal battery behavior but rather pseudocapacitance-like behavior.

**Figure 4:**
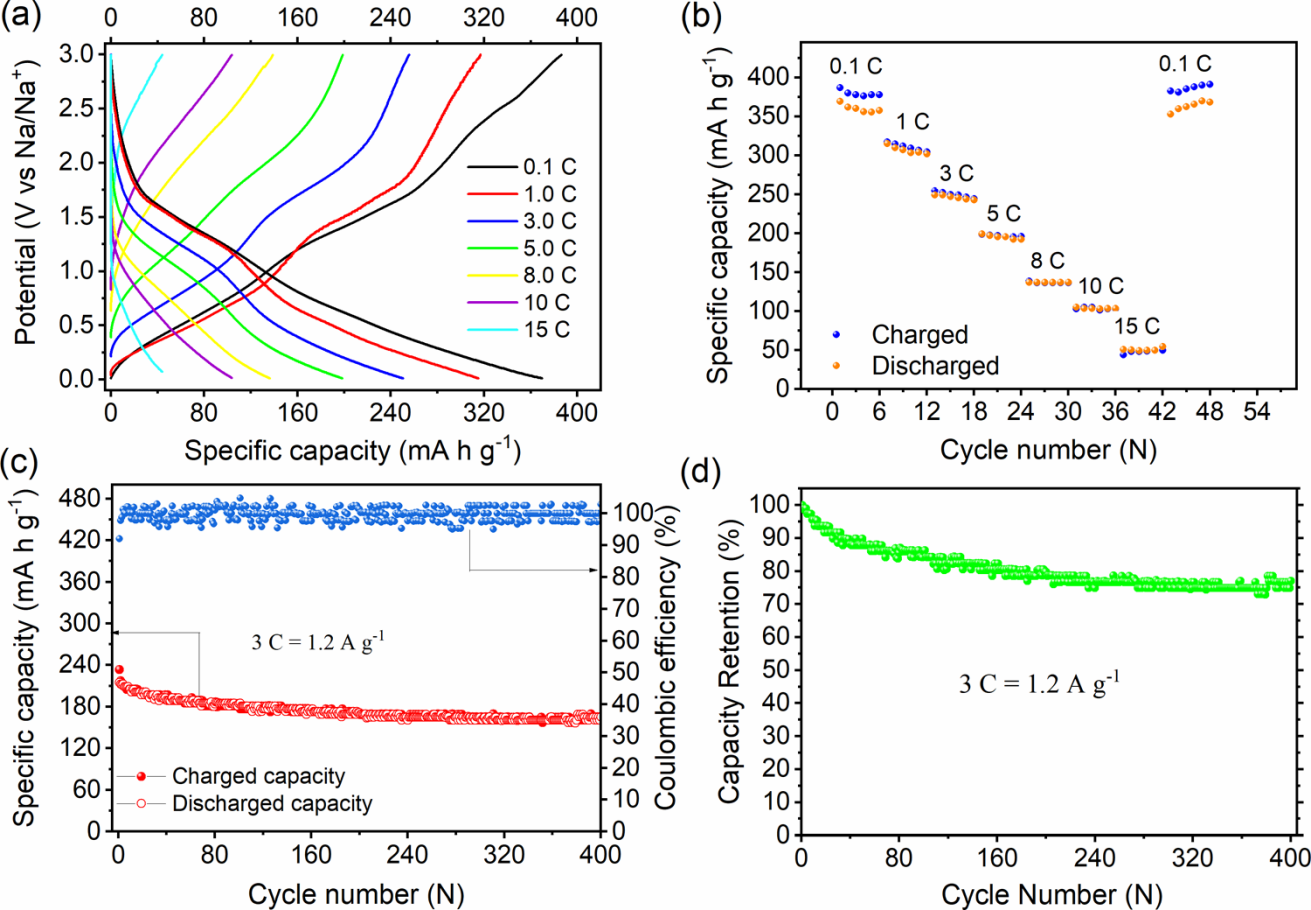
(a) Galvanostatic charge/discharge plots at different C rates. (b) Rate capability at different current rates varies from 0.1 to 15 C. (c) Cycling stability and Coulombic efficiency over 400 cycles. (d) Capacity retention at 3 C over 400 cycles.

Based on the impedance data generated by Randles equivalent circuits, the conductivity of the BCOF-1 electrode based on the total resistance (*R*_ct_, *R*_el_, and *R*_w_) was calculated to be 5.13 × 10^–3^ S m^–1^ for the fresh coin cell, which can be attributed to the small diffusion resistance and swift Faradaic charge transfer *R*_ct_. On the other hand, the 400-cycled coin cell showed a lower conductivity of 1.96 × 10^–4^ S m^–1^ due to a slower charge transfer and sluggish ion diffusion built upon cycling (SI, Figure S24).^59^ The BCOF-1-based electrode performance was examined by its rate capability and long-term cycling stability. The electrode showed a stable reversible discharge specific capacity of 362, 311, 249, 196, 136, 104, 50 mA h g^–1^ at current rates of 0.1, 1, 3, 5, 8, 10, and 15 C, respectively. As the current rate was decreased back to 0.1 C, the discharge-specific capacity of 365 mA h g^–1^ was reattained with 100% capacity recovery and proved the fast charge exchange kinetics during the charge and discharge process (Figure 4a,b). Furthermore, the capacity drop from 0.1 to 1 C was only 14%, which is very modest after a 10-fold current rate increase. The excellent rate capability performance could be attributed to the high surface area, high electronic conductivity, and the honeycomb-like porous channels of BCOF-1.^36^ Charge/discharge hysteresis was clearly observed at 0.1 C because of the Na^+^ ion depletion within the pores in the inner regions caused by the lower diffusion rate compared to that in the outermost areas of the BCOF-1 framework. This could restrict accessibility to the aza-active sites by the Na^+^ ions.^63^ On the other hand, all current rates higher than 0.1 C demonstrated outstanding reversibility by showing no hysteresis between the charge and discharge processes. The rate capability figure showed the capacity drop at higher current rates, which is attributed to the fast Na^+^ ion diffusion and thus lowered the chance of reaching the redox-active sites.^64^ To this end, we have utilized the impedance data to estimate the diffusion coefficient of Na^+^ ions (*D*_Na_^+^) before (1.09 × 10^–12^ cm^2^ s^–1^) and after being cycled (2.19 × 10^–14^ cm^2^ s^–1^) (SI, Figure S25a,b).

The long-term cycling stability of the BCOF-1-based electrode was performed over 400 cycles. The electrode demonstrated excellent stability with an outstanding Coulombic efficiency of nearly 100% at 3 C (Figure 4c). Furthermore, the electrode also exhibits good capacity retention after 400 charge/discharge cycles of ∼ 77%. It is worth mentioning that the largest capacity drop took place during the first 100 cycles, which represents 70% of the total capacity drop (Figure 4d). The porous π-conjugated and insoluble nature of the BCOF-1 framework presumably plays an essential role in the cycling stability of the electrode by retarding framework dissolution in the electrolyte.^36^

**Figure 5:**
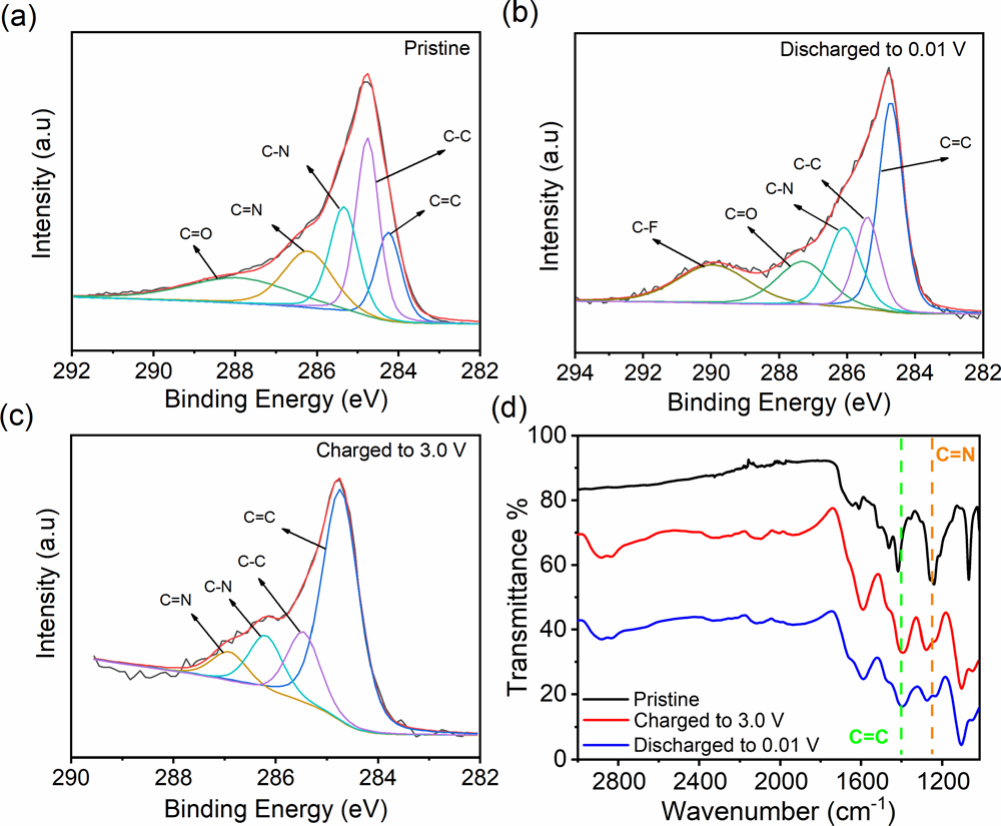
Deconvoluted C 1s XPS spectra of BCOF-1 at different stages: (a) pristine, (b) discharge to 0.01 V, (c) charged to 3.0 V, and (d) ATR-IR for sodiation/desodiation mechanism investigation.

The redox mechanism (charge/discharge) of BCOF-1-based electrodes was monitored by X-ray photoelectron spectroscopy (XPS) and ATR-IR (Figure 5) during the sodiation and desodiation processes at three different stages: pristine, charged, and discharged. XPS and IR results indicate the disappearance of the C=N pyrazine ring band at 286.6 eV and 1246 cm^–1^, respectively, when the electrode was discharged to 0.01 V, indicating the formation of C–N–Na (Figure 5b,d). However, the C=N band was reinstated when the electrode was recharged to 3.0 V, confirming the high redox reversibility of the aza centers (Figure 5c). Furthermore, IR results show that the C=C bonds remain redox inactive and do not contribute to the overall capacity; this observation was supported by the C=C band positions at 1412 and 1600 cm^–1^, which stayed intact in all three stages (pristine, charged, and discharged), indicating that aza redox-active sites are the only contributor to the total capacity (Figure 5d).

The high porosity and well-aligned 1D channels of BCOF-1 are expected to play important roles in accessing high energy and power density. Energy density and power density calculations were conducted to investigate the efficiency of the BCOF-1-based electrode with regard to energy and power per unit mass. At a current rate of 0.1 C, the BCOF-1 electrode demonstrated a remarkable energy density of 302 W h kg^–1^ (SI, Figure S26a), which is comparable to the best-performing inorganic and organic electrodes used in SIBs.^8,46,65^ Furthermore, even at a high C rate of 5 C, BCOF-1 still delivers a specific energy of 136 W h kg^–1^, which corresponds to a remarkable power density of 682 W kg^–1^ (SI, Figure S26b).

## Conclusions

In this work, we developed a simple synthetic route for the synthesis of multifunctional benzimidazole-linked COFs and demonstrated their potential application in sodium-ion batteries. The highly porous and π-conjugated nature of BCOF-1 facilitates rapid sodium ion diffusion and access to the redox-active aza sites, leading to high specific capacity, long cycling stability, and superior rate capability. BCOF-1 exhibits a pseudocapacitive-like behavior, which is essential for accessing both high energy and power density in batteries. This study demonstrates that BCOFs have the desired electrochemical and textural properties to advance the application of organic materials as anodes in sustainable sodium-ion batteries. We expect the reactive pore surface at the imidazole sites in BCOFs to provide additional means for regulating ion transport through the 1D channels, and we aim to explore this area in our future studies.

## Supporting Information Available

Experimental details, ATR-IR, PXRD, PSD, CV, TGA, AFM, SEM, Raman spectral data of BCOF-1, structural studies, and unit cell parameters from modeling and simulation studies (PDF)
