# Supplementary material for: High Sodium Ion Storage by Multifunctional Covalent Organic Frameworks for Sustainable Sodium Batteries
Source: ACS Appl Mater Interfaces. 2024 Mar 18;16(12):14750–8. doi: 10.1021/acsami.3c17710 (PMC10982936; doi:10.1021/acsami.3c17710)
Supplement: Supplementary file 1 — am3c17710_si_001.pdf [file am3c17710_si_001.pdf]

Supporting Information

High Sodium Ion Storage by Multifunctional Covalent Organic Frameworks for Sustainable Sodium Batteries

Mohammad K. Shehab and Hani M. El-Kaderi*

Department of Chemistry, Virginia Commonwealth University, Richmond, VA, 23284, United States

*Corresponding author: E-mail: helkaderi@vcu.edu

Table of Contents

	Page
Experimental Section: Synthesis procedure, Schemes, NMR and ATR-IR	*S3-S19*
Crystallographic parameters for AA stacking BCOF-1	*S20*
Crystallographic parameters for AB stacking BCOF-1	*S20*
The N_2 adsorption/desorption isotherm of BCOF-1	*S21*
The pore size distribution of BCOF-1	*S21*
SEM images of as-prepared BCOF-1	*S21*
Cyclic voltammograms of BCOF-1 monomer electrode in 1M $NaPF_6$/ DEGDME at a scan rate of 0.1 mV s^{-1} of the potential range between 0.01 and 3.0 V	*S22*
Cyclic voltammogram of BCOF-1 electrode for cycle 6 at a scan rate of 0.1 mVs^{-1}	*S22*
Electrochemical impedance spectra for a) battery before and after 400 cycles	*S23*
Simple equivalent Randles circuit diagram for the coin cells before and after cycling	*S23*
BCOF-1 Electrode Film thickness measurement	*S23*
The plot of the real impedance resistance, Z′, vs. the reciprocal root square of the lower angular frequencies ($\omega^{-0.5}$) for a fresh battery and after 400 cycles	*S24*
The energy density of BCOF-1 at different current rates of 0.1, 1, 3, 5, 8, 10, and 15	*S24*
Ragone plot for BCOF-1 based electrode at 0.1, 1.0, 3.0, and 5.0 C.	*S24*
Thermal gravimetric analysis of BCOF-1	*S25*
Diffusion coefficient calculations	*S25*
Energy density and power density calculations	*S26*
Comparison of the sodium-storage performance of BCOF-1 with representative covalent organic frameworks SIB electrodes. (AM = active material, PVDF = Polyvinylidene fluoride)	*S27-28*
References	*S29-30*

Experimental Section

Scheme 1. Overall synthesis of HATNHA.

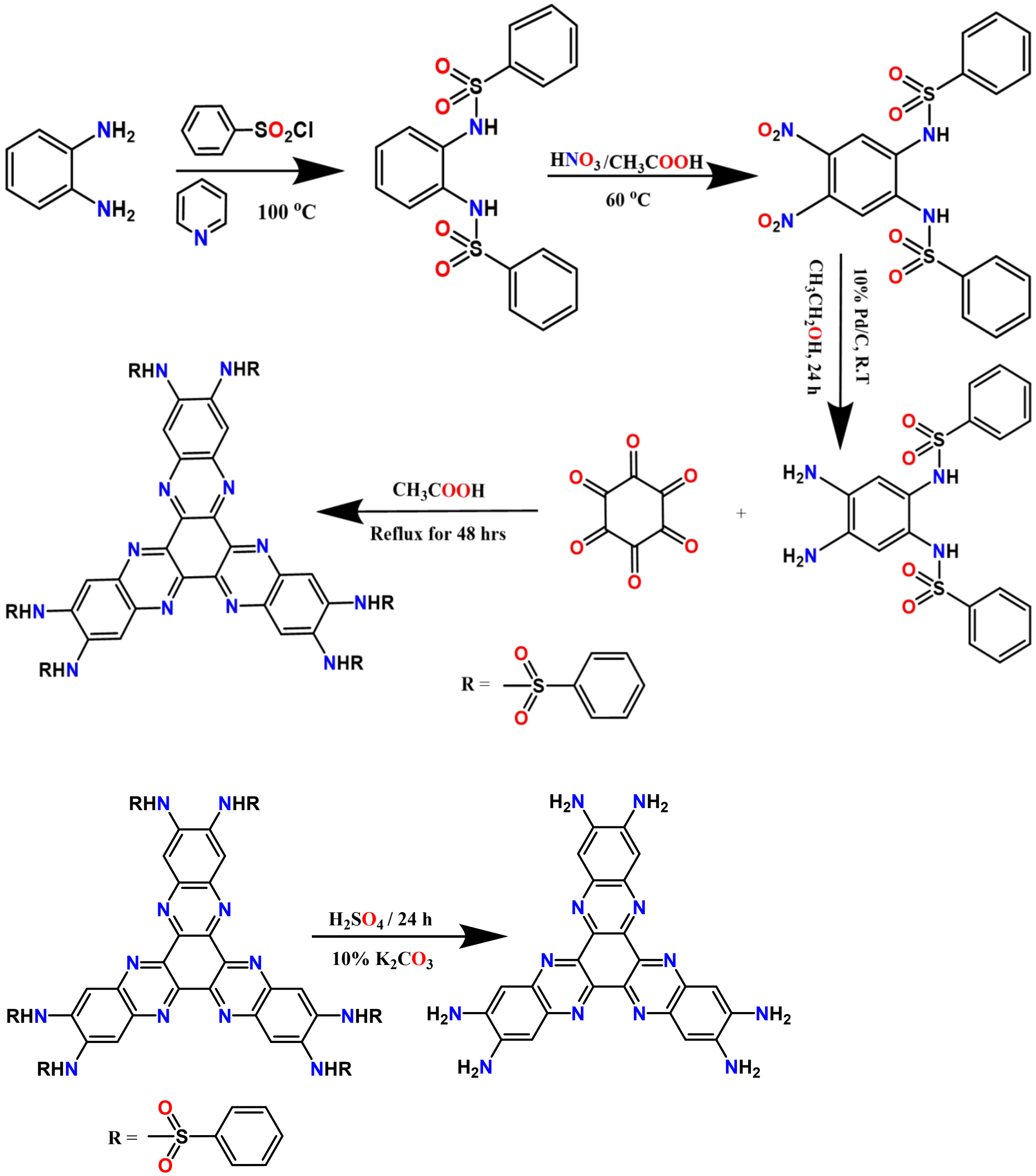

1,2-Bis-(phenylsulfonamido) benzene[1]

20 g of 1,2-diaminobezene (185 mmol) and 65 ml pyridine were charged in 250 ml 2-neck flask under nitrogen. A solution of 47.20 ml benzenesulfonyl chloride (370 mmol) and 10 ml pyridine was added slowly into the flask. The solution mixture was heated for 5 hours at 100 ℃ under nitrogen. The solution was then poured into 250 ml water and stirred for 30 seconds to get a pale yellow precipitate. The crude product was recrystallized with ethyl acetate by slow evaporation at room temperature to afford off-white crystals and then dried at 110 ^oC. (>90% yield) ATR-IR; 3272 cm^{-1} for (NH), 3070 cm^{-1} for (=C-H), 1597 cm^{-1} for (C=C), 1153 and 1331 cm^{-1} for (S=O). ^1H NMR (400 MHz, DMSO-d6) δ 6.986 (t, 2H), 6.989 (t, 2H), 7.55 (t, 4H), 7.66 (t, 2H), 7.72 (d, 4H), 9.35 (s, 1H); ^{13}C NMR δ 123.91, 126.39, 127.31, 129.79, 130.17, 133.72, and139.48 ppm.

Scheme S2. The synthesis of 1,2-Bis-(phenylsulfonamido) benzene from 1,2-diaminobenzene.

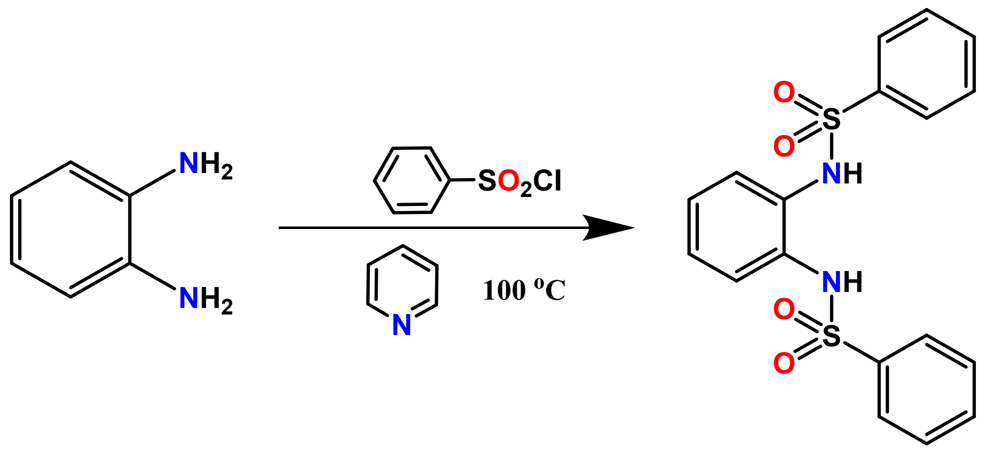

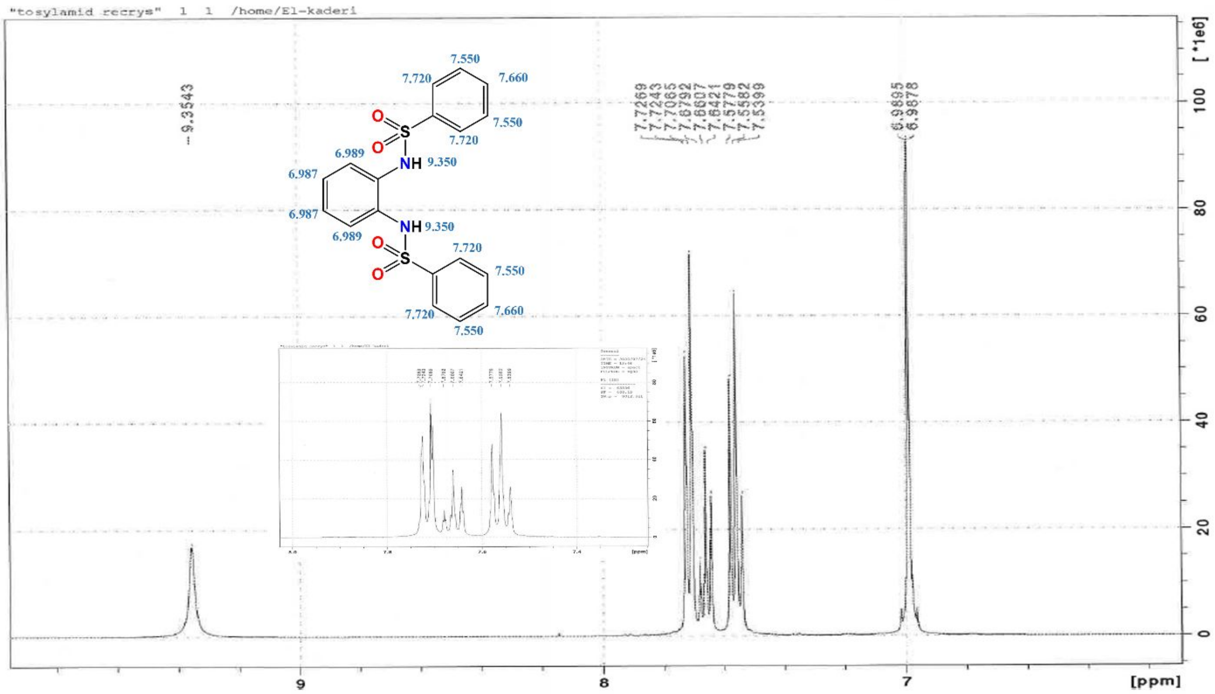

Figure S1. ^{1}HNMR of 1,2-Bis-(phenylsulfonamido) benzene.

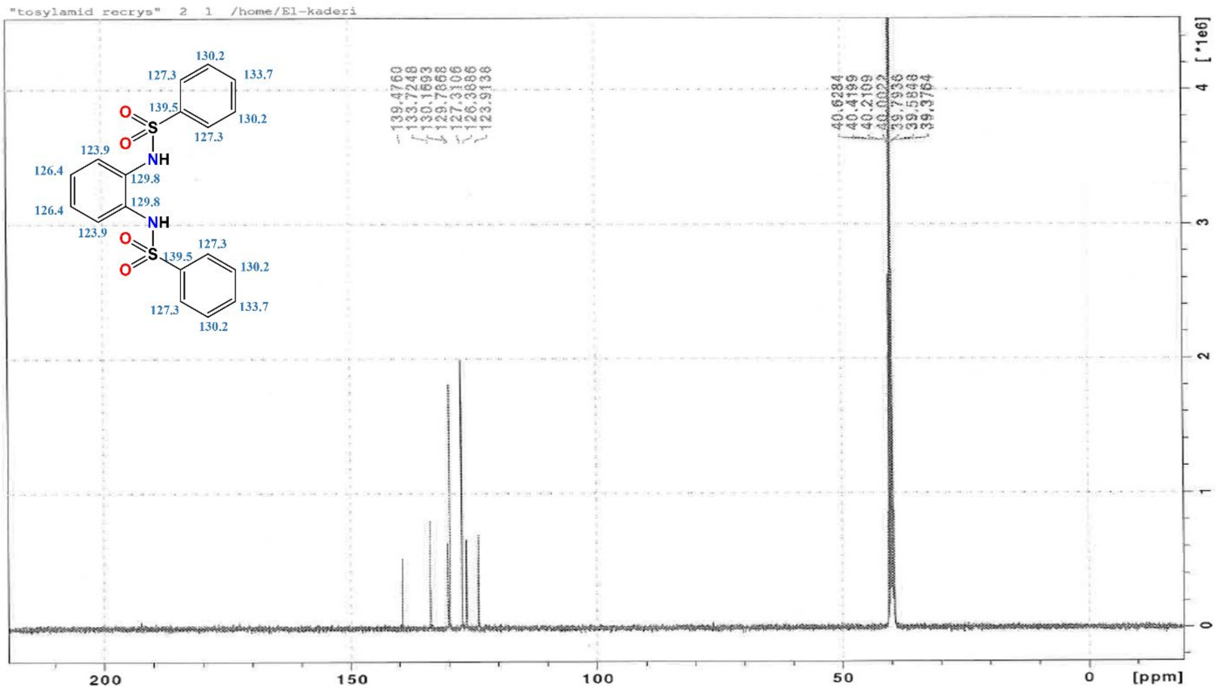

Figure S2. ^{13}CNMR of 1,2-Bis-(phenylsulfonamido) benzene.

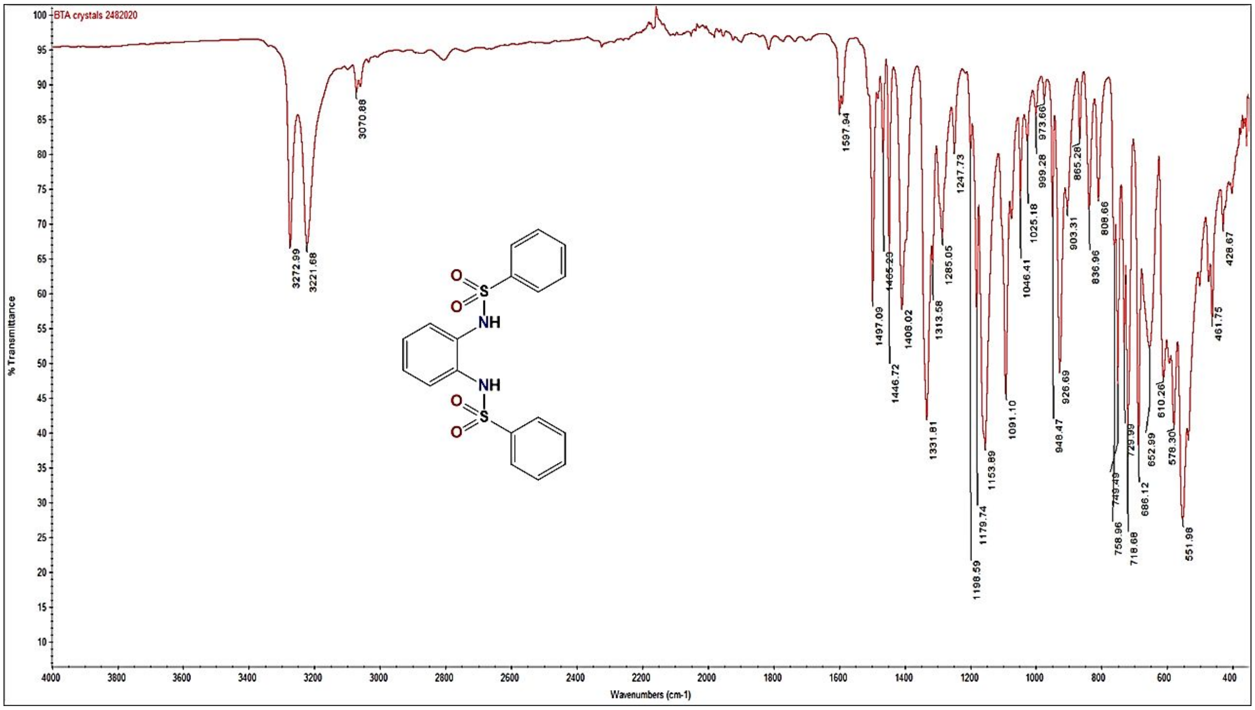

Figure S3. ATR-IR of 1,2-Bis-(phenylsulfonamido) benzene.

1,2-Bis-(phenylsulfonamido)-4,5-bis-nitrobenzene[2]

20 g of 1,2-Bis-phenyl-sulfonamido-benzene (51 mmol) was put in a 500 ml two-neck flask, and 175 ml CH_3COOH was added. Then a mixture of acids (6.00 ml fuming HNO_3 and 7.00 ml CH_3COOH) was added dropwise at 60 ^{o}C with stirring. After the addition was completed, the mixture solution was reacted again for 30 minutes at 60 ^{o}C, cooled, filtrated, and obtained shallow yellow solid. The product was washed with acetic acid, recrystallized from ethanol by slow evaporation at room temperature to afford off-white needle crystals, and dried in the oven at 110 ^{o}C; ATR-IR; 3265 (NH), 3071 cm^{-1} (=C-H), 1593 cm^{-1} (C=C)1357 and 1529 cm^{-1} (NO_2), 821 cm^{-1} (Ar-NO_2), 1163 and 1331 cm^{-1} (S=O).^{1}HNMR (400 MHz DMSO-d6), 8.83 (s, 2H), 7.79 (d, 4H), 7.73 (s, 2H), 7.67(t, 2H), 7.57 (t, 4H) ; ^{13}C NMR δ 115.80, 127.23, 127.31, 129.99, 133.98, 134.92, 137.15 and 139.64 ppm.

Scheme S3. The synthesis of 1,2-Bis-(phenylsulfonamido)-4,5-bis-nitrobenzene.

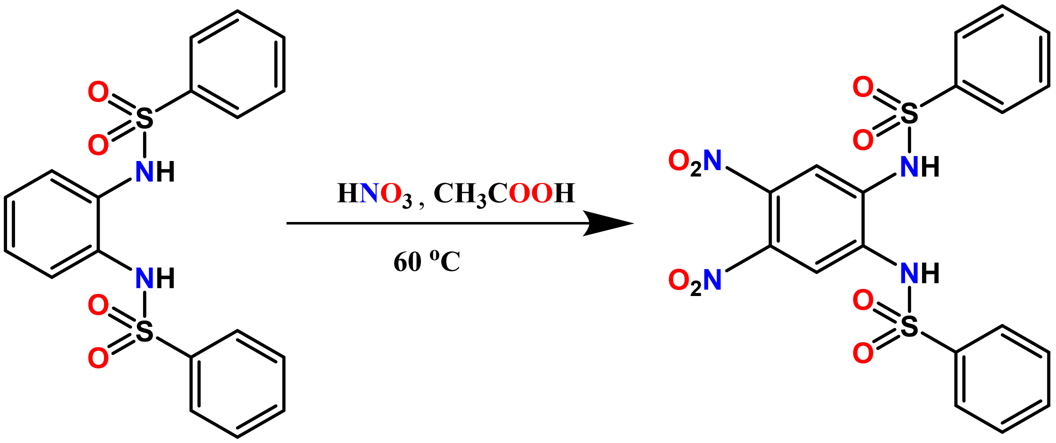

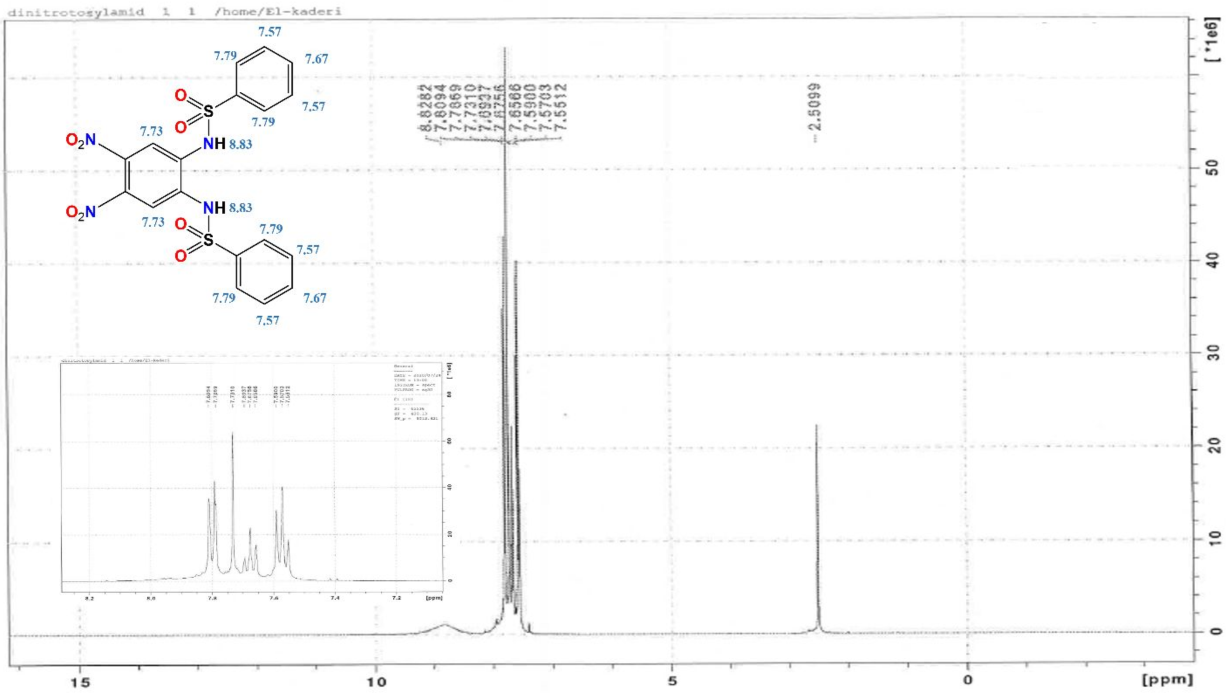

Figure S4. ^{1}HNMR of 1,2-Bis-(phenylsulfonamido)-4,5-bis-nitrobenzene.

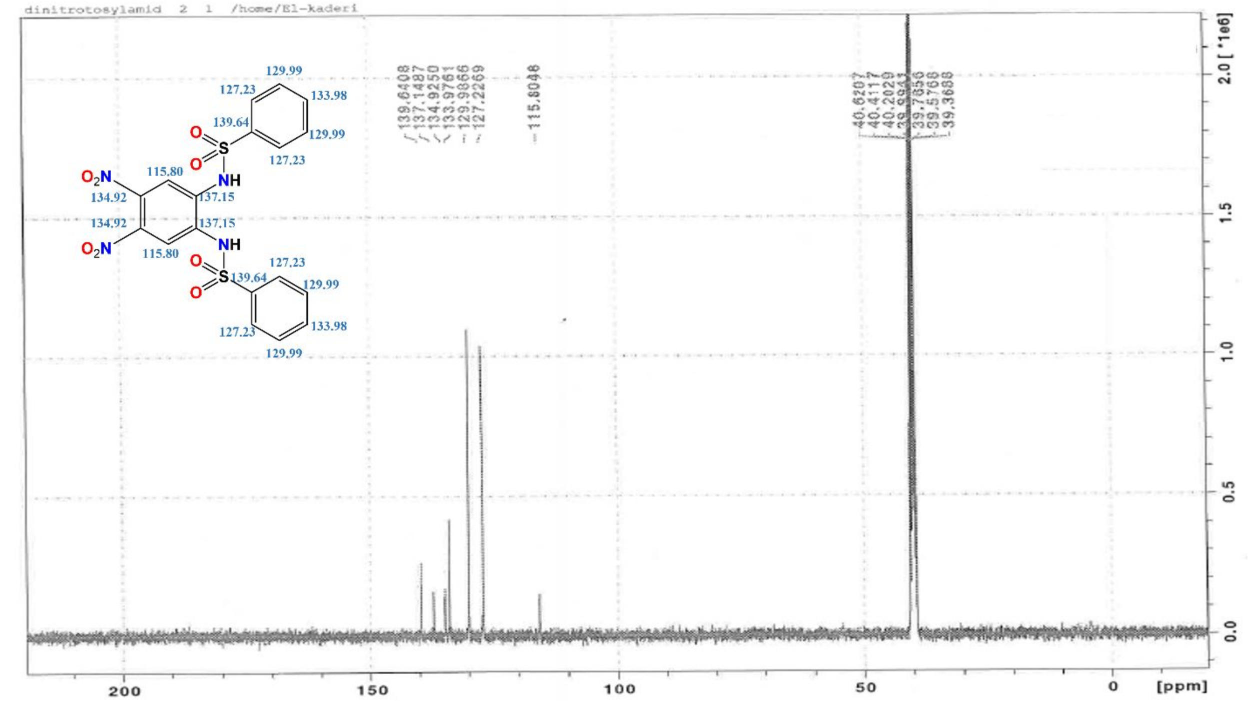

Figure S5. ^{13}CNMR of 1,2-Bis-(phenylsulfonamido)-4,5-bis-nitrobenzene.

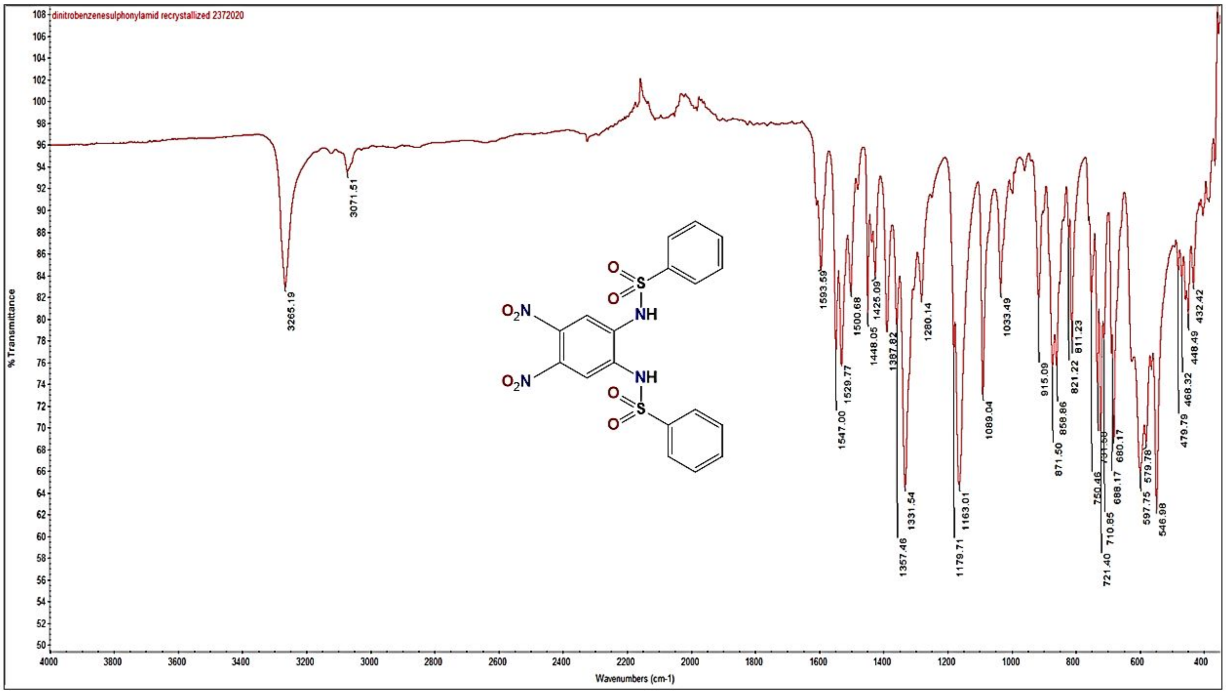

Figure S6. ATR-IR of 1,2-Bis-(phenylsulfonamido)-4,5-bis-nitrobenzene.

1,2-Bis-(phenylsulfonamido)-4,5-bis-aminobenzene[3]

A 100 ml high-pressure vessel reactor was charged with 5.0 g of N,N'-(4,5-Dinitro-1,2-phenylene)dibenzenesulfonamide (10.5 mmol), 60.0 ml an ethyl acetate, 60 mg 10% Pd/C powder, and a stir bar. H_2 gas at high pressure (60 psi) was provided to the vessel, and the mixture was under stirring for 24 hours at room temperature. The mixture was filtered using a frit funnel and washed with boiling ethanol (2x100 ml each), and the filtrate was let to cool down at room temperature and then in an ice bath to afford off-white DPDS crystals (yield >90%). ATR-IR; 3270 (NH, sulfonamide), 3367 and 3436 cm^{-1}(NH_2 symmetrical and asymmetrical stretching), 3071 cm^{-1} (=C-H), 1629 cm^{-1} (C=C), 1158 and 1323 cm^{-1} (S=O).1H NMR (400 MHz, DMSO-d6) δ 4.53 (s, 2H), 6.13 (s, 2H),7.53 (t, 4H), 7.62 (t, 2H), 7.64 (d, 4H), 8.57 (s, 2H); ^{13}C NMR δ 111.34, 120.43, 127.32, 129.47, 133.28, 134.22, and 139.61 ppm.

Scheme S4. The synthesis of 1,2-Bis-(phenylsulfonamido)-4,5-bis-aminobenzene.

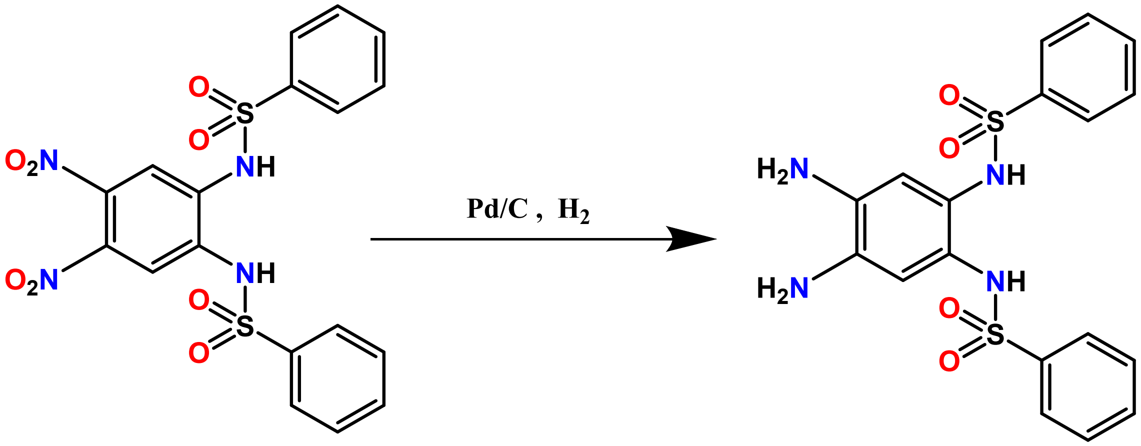

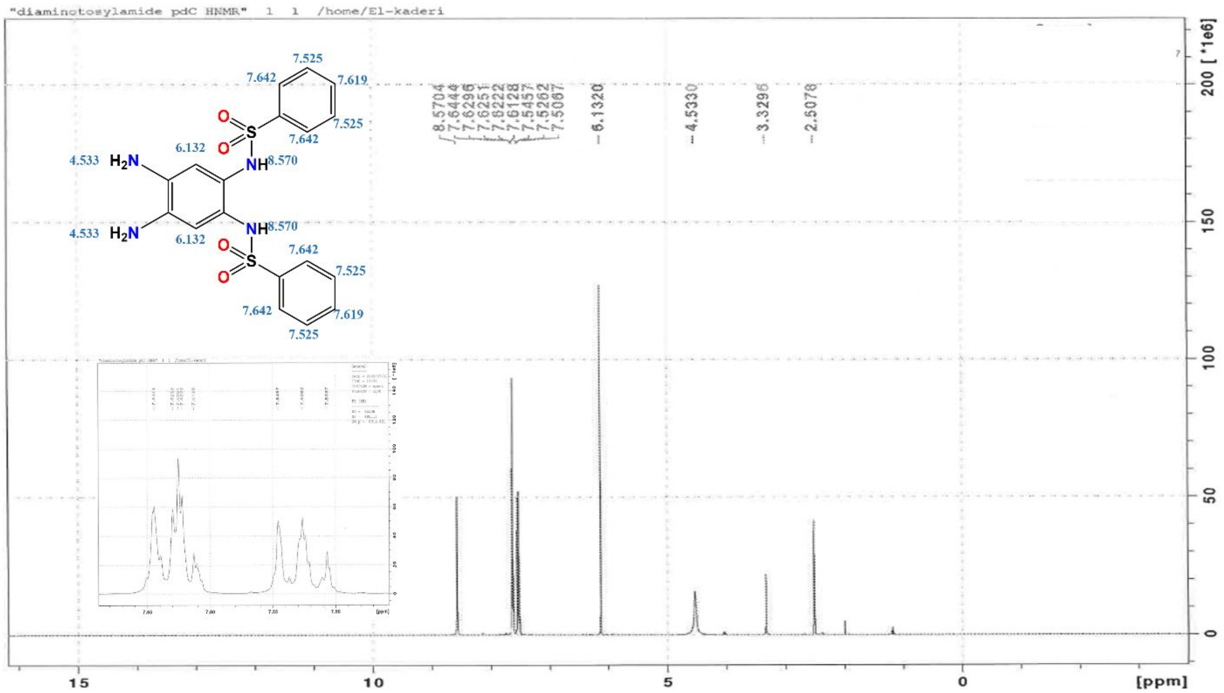

Figure S7. ^{1}HNMR of 1,2-Bis-(phenylsulfonamido)-4,5-bis-aminobenzene.

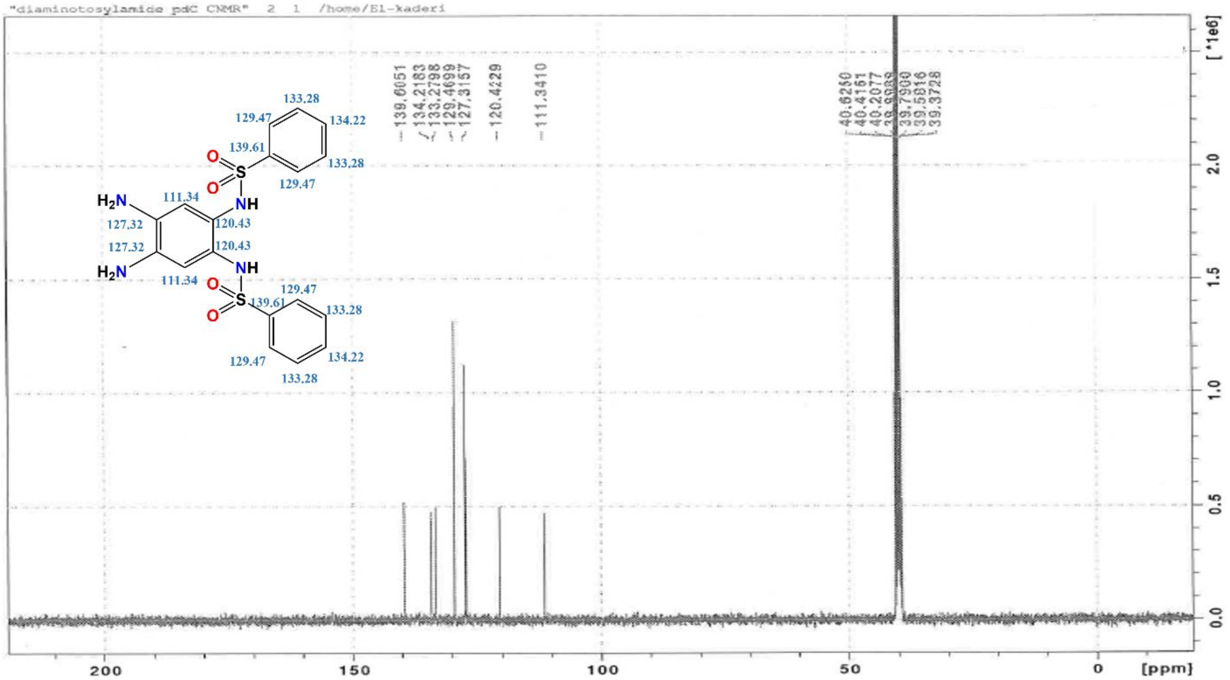

Figure S8. ^{13}CNMR of 1,2-Bis-(phenylsulfonamido)-4,5-bis-aminobenzene

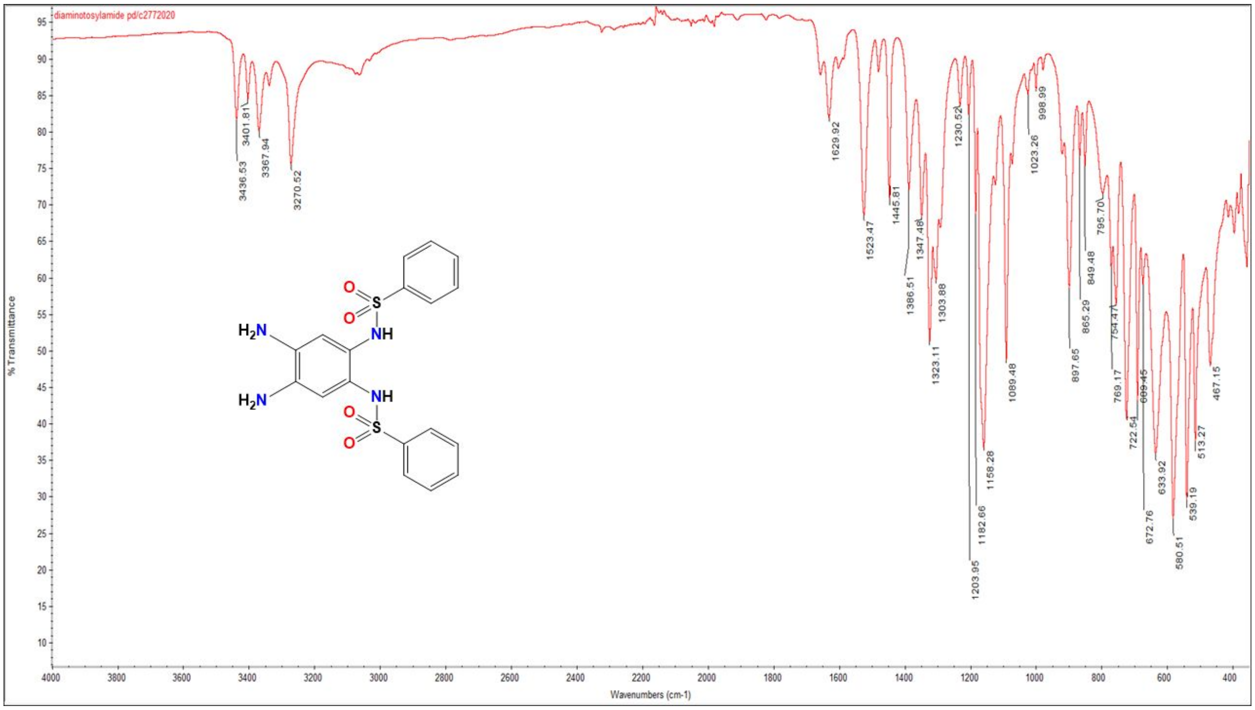

Figure S9. ATR-IR of 1,2-Bis-(phenylsulfonamido)-4,5-bis-aminobenzene

<u>Hexaazatrinaphthalenehexasulfonamide (HATNHSA)</u>[4]

1.0 g of 4,5-Diamino-1,2-phenylene-dibenzenesulfonamide (2.39 mmol) and 248.8 mg of hexaketocyclehexane octahydrate (0.80 mmol) was mixed with 25 ml deoxygenated acetic acid in a 100 ml three-necked round bottom flask. The mixture was heated for 24 hours at 120 ^{o}C. After cooling down to room temperature, the solution was poured into a 500 ml water-ice slurry. Then, filter out the green precipitate using a frit funnel. The crude product was air-dried to afford a green precipitate with a high yield. The crude product was stirred in hot ethanol for 10 minutes for further purification, and the solution was filtered while it was warm. The product was dried in the oven overnight at 90 °C. (>90 % yield) ATR-IR; 3297 (NH), 3071 cm^{-1} (=C-H), 1622, 1479 cm^{-1} (C=C), 1220 cm^{-1} (C=N), 1156 and 1328 cm^{-1} (S=O). ^{1}H NMR (400 MHz, DMSO-d6) δ 7.62 (t, 12H), 7.70 (t, 6H), 7.94 (d, 12H), 7.99 (s, 6H); ^{13}C NMR δ 118.5, 127.4, 130.2, 134.2, 135.0, 139.4, 140.1, 143.5 ppm.

Scheme S5. The synthesis of HATNHSA.

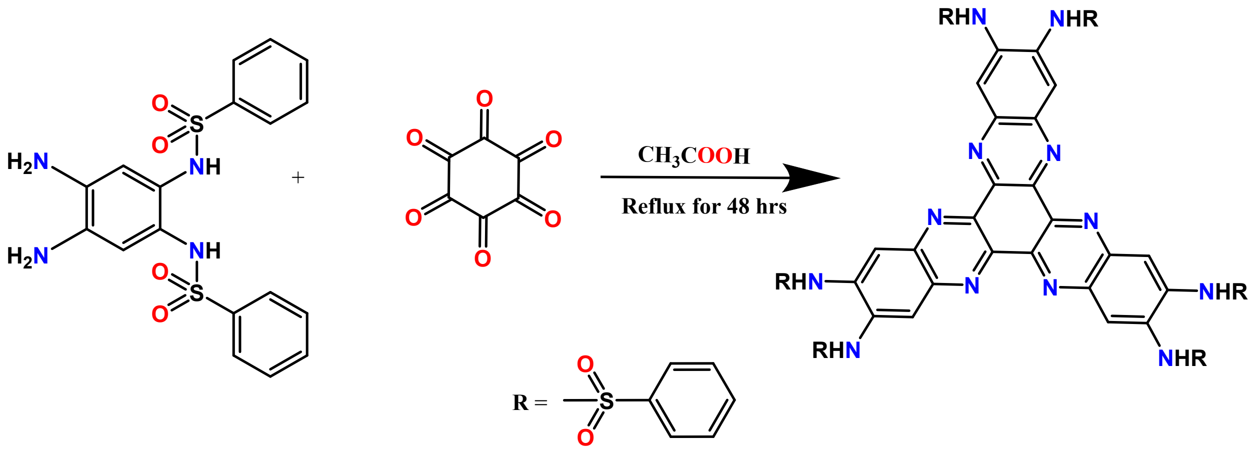

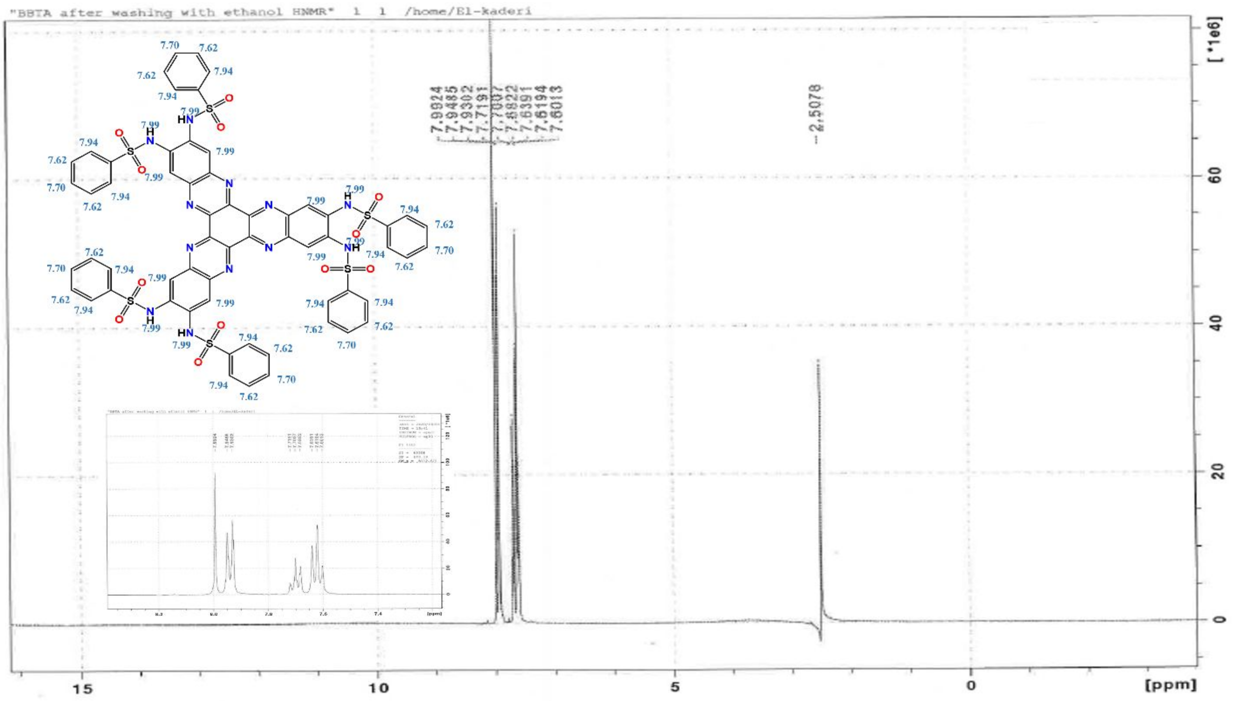

Figure S10. ^{1}HNMR of HATNHSA.

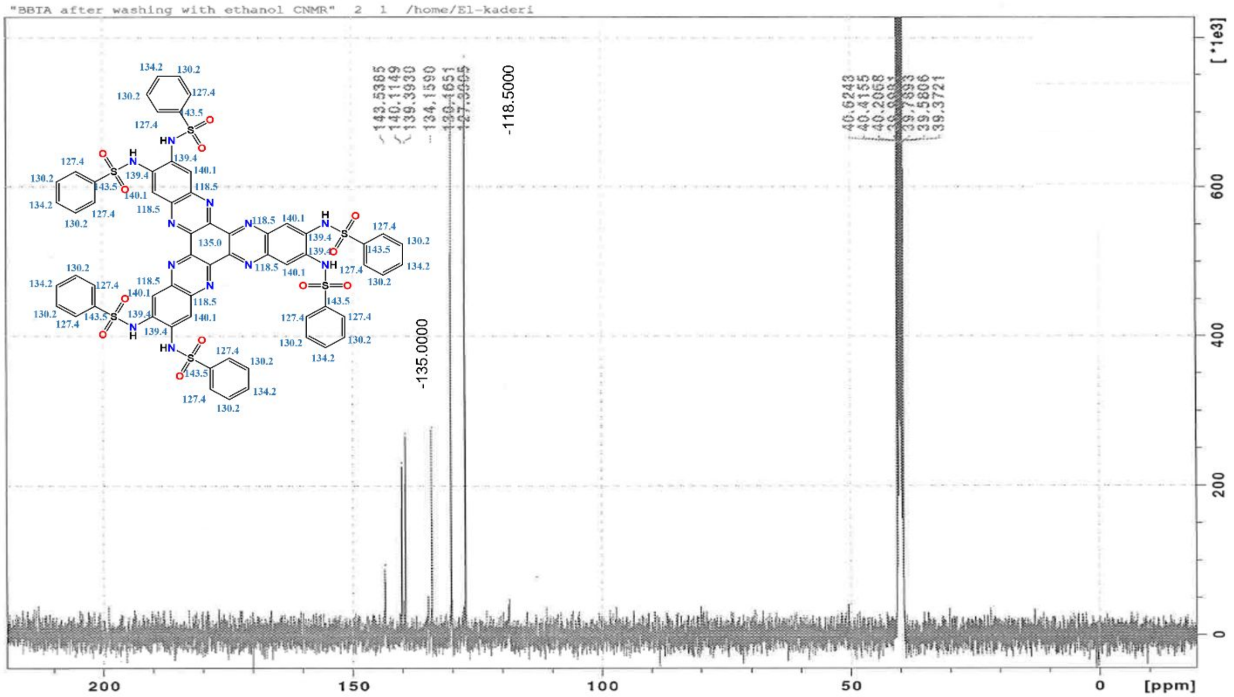

Figure S11. ^{13}CNMR of HATNHSA.

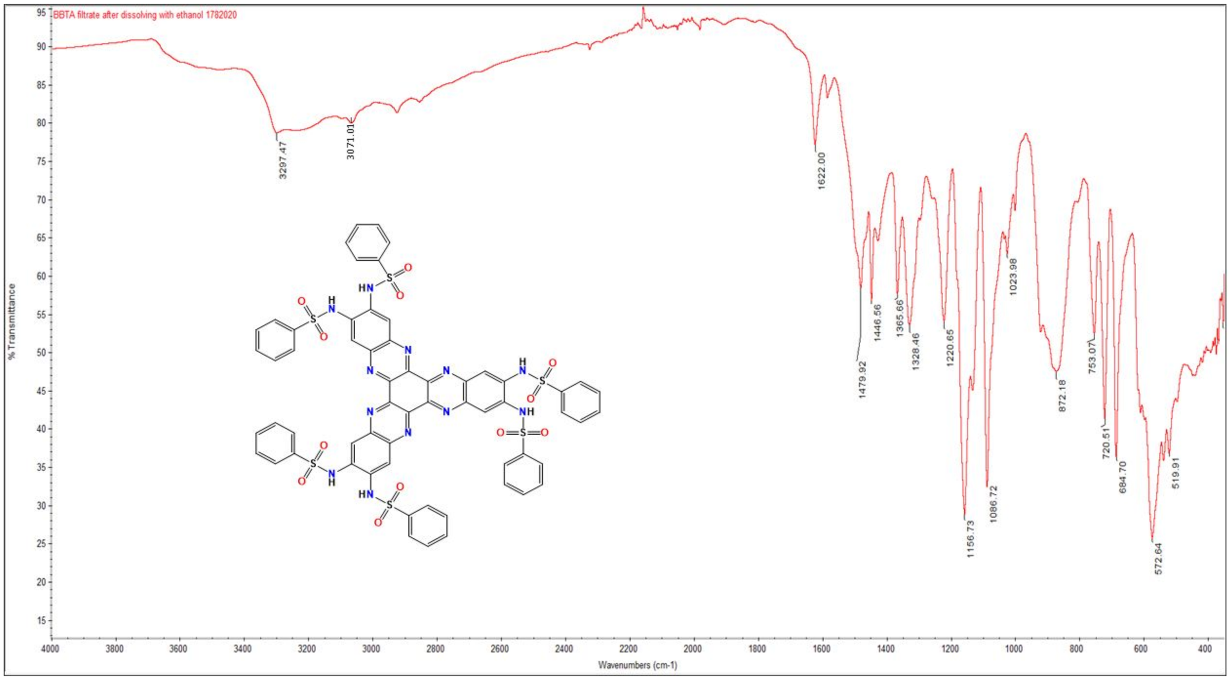

Figure S12. ATR-IR of HATNHSA.

Hexaazatrinaphthalenehexamine (HATNHA)[5]

1.0 g of HATNHSA (0.76 mmol) was mixed with 25 ml conc. H_2SO_4 in 100 ml round bottom flask and stirred for 24 h at room temperature. After 24 hours, the solution was poured into 1000 g of ice to avoid the exothermic heat between H_2SO_4 and water. When the ice has melted, the precipitate was filtered out and washed with distilled water (30 ml x 3). Then, the precipitate was collected and dispersed in the 10% K_2CO_3 solution and stirred for 3 hours. After completion, the product was filtered out, washed many times with double deionized water (DDW) (30 ml x 5) and recrystallized with DMSO/water, the product was collected and dried under a vacuum to afford shiny dark green building block HATNHA. (93% yield) ATR-IR; 3184 and 3275 (NH_2 symmetrical and asymmetrical stretching), 3071 cm^{-1} (=C-H), 1634, 1492 cm^{-1} (C=C), 1262 cm^{-1} (C=N). ^1H NMR (400 MHz, DMSO-d6) δ 6.14 (s, 12H), 7.23 (s, 6H); 13C NMR δ 105.09, 139.25, 140.27, 143.40 ppm.

Scheme S6. The synthesis of HATNHA.

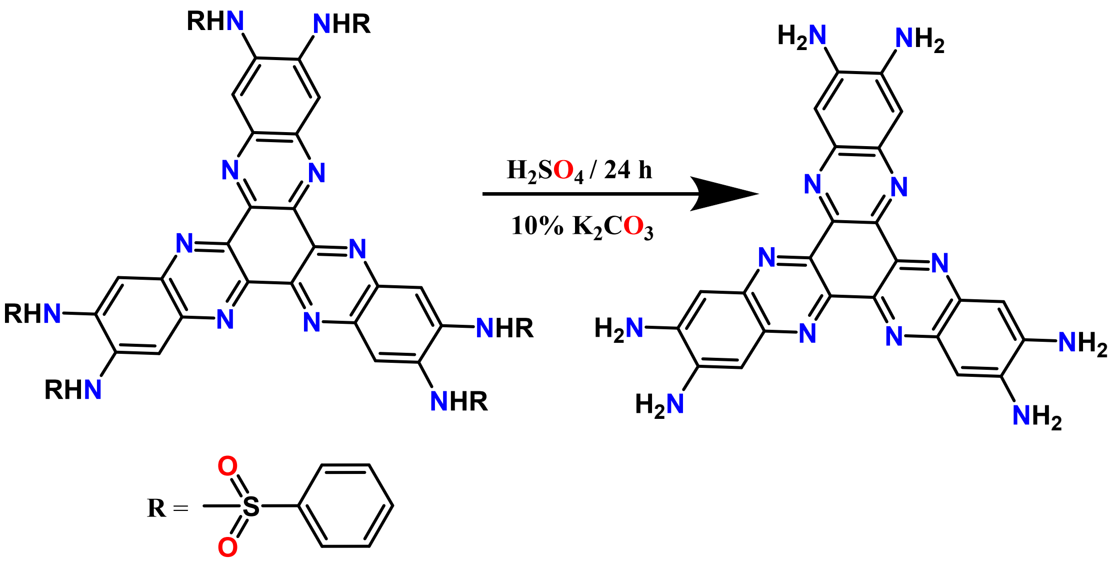

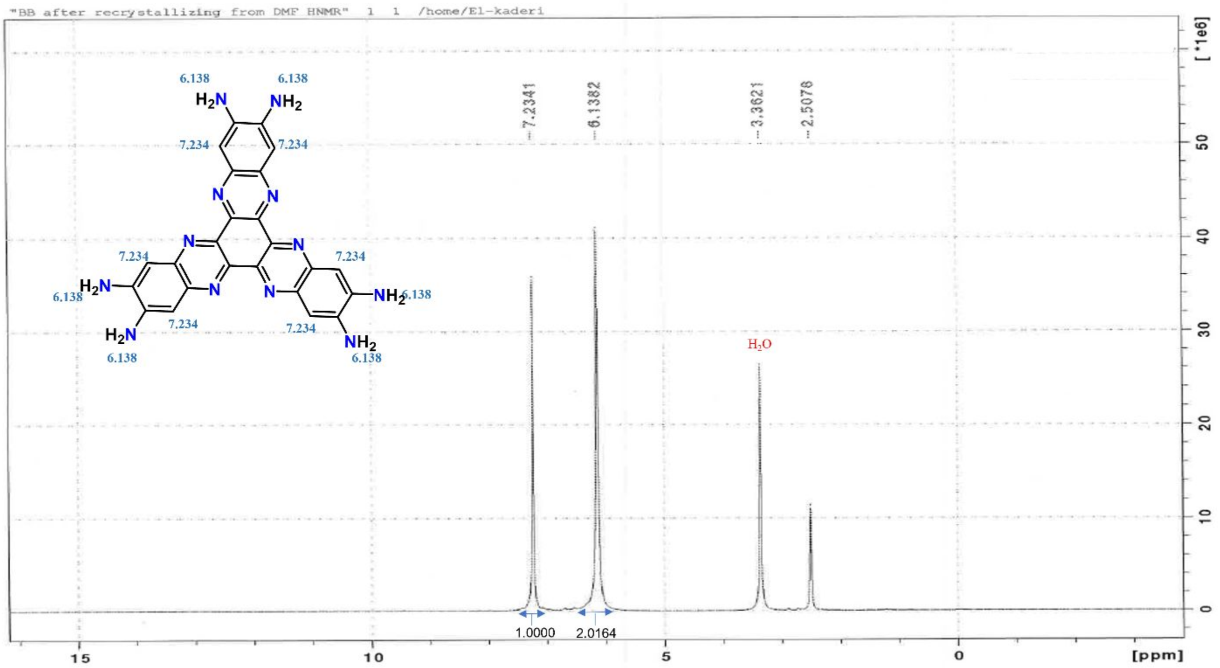

Figure S13. ^{1}HNMR of HATNHA.

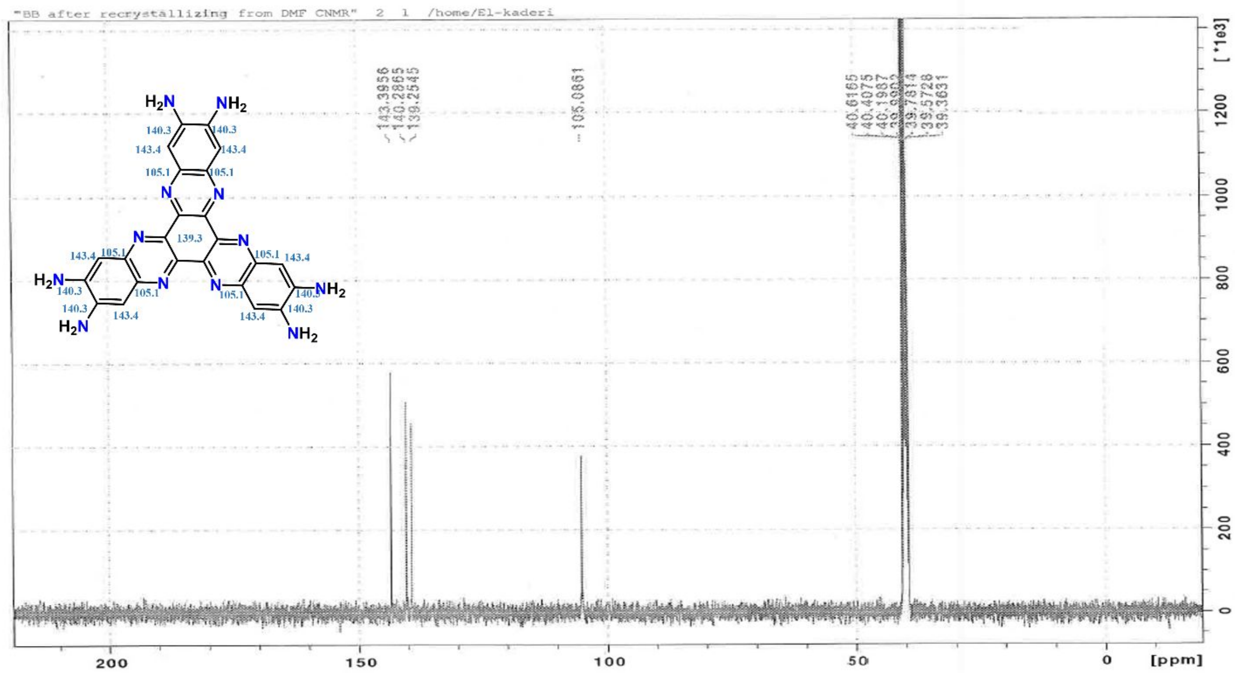

Figure S14. ^{13}CNMR of HATNHA.

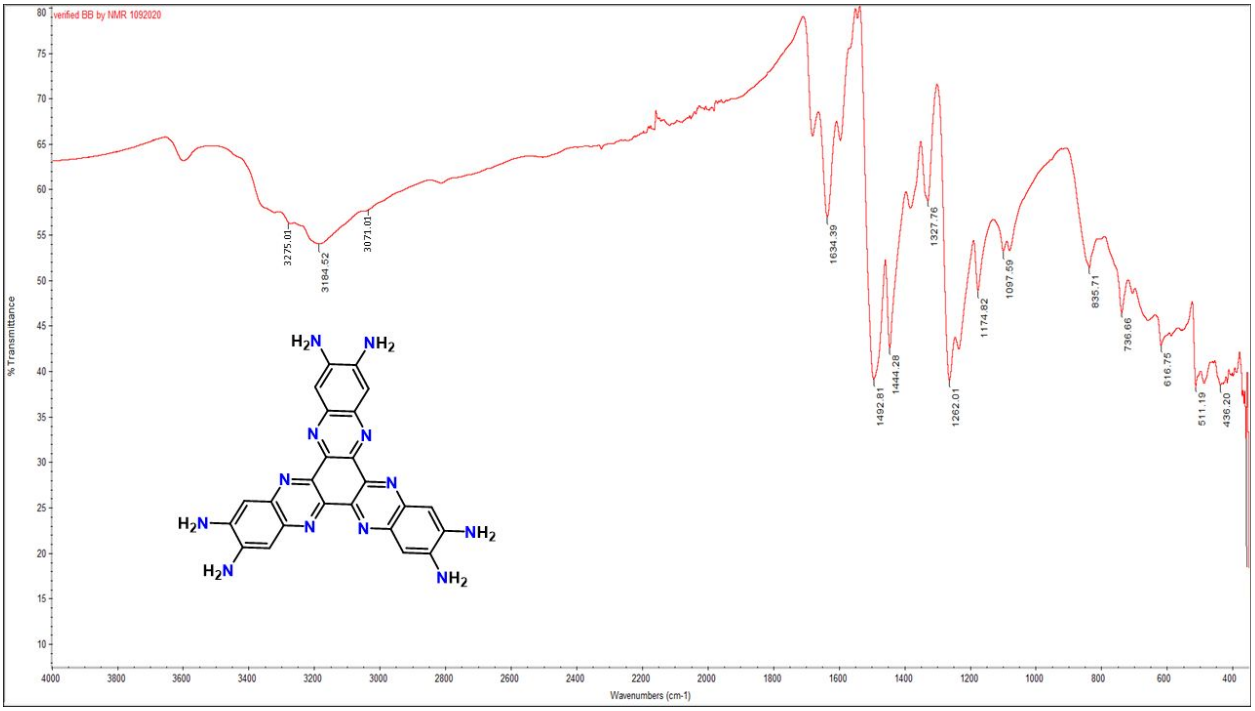

Figure S15. ATR-IR of HATNHA.

Synthesis of BCOF-1 monomer

HATNHA (100 mg, 0.210 mmol) and benzaldehyde (67.0 mg, 64.4 µl, 0.632 mmol) was mixed with 25 ml deoxygenated acetic acid in a 100 ml three-necked round bottom flask. The mixture was heated for 48 hours at 120 ^{o}C. After cooling down to room temperature, the solution was poured into a 500 ml water-ice slurry. Then, the product was filtered out using a frit funnel to afford black precipitate with a good yield. ATR-IR, 3138 cm^{-1} (N-H stretching), 3071 cm^{-1} (=C-H), 1632, 1410 cm^{-1} (C=C), 1247 cm^{-1} (C=N). ^{1}H NMR (400 MHz, DMSO-d6) δ 7.23 (m, 15H), 7.82 (s, 6H), 12.6 (s, 3N-H).

Scheme S7. The synthesis of BCOF-1 monomer.

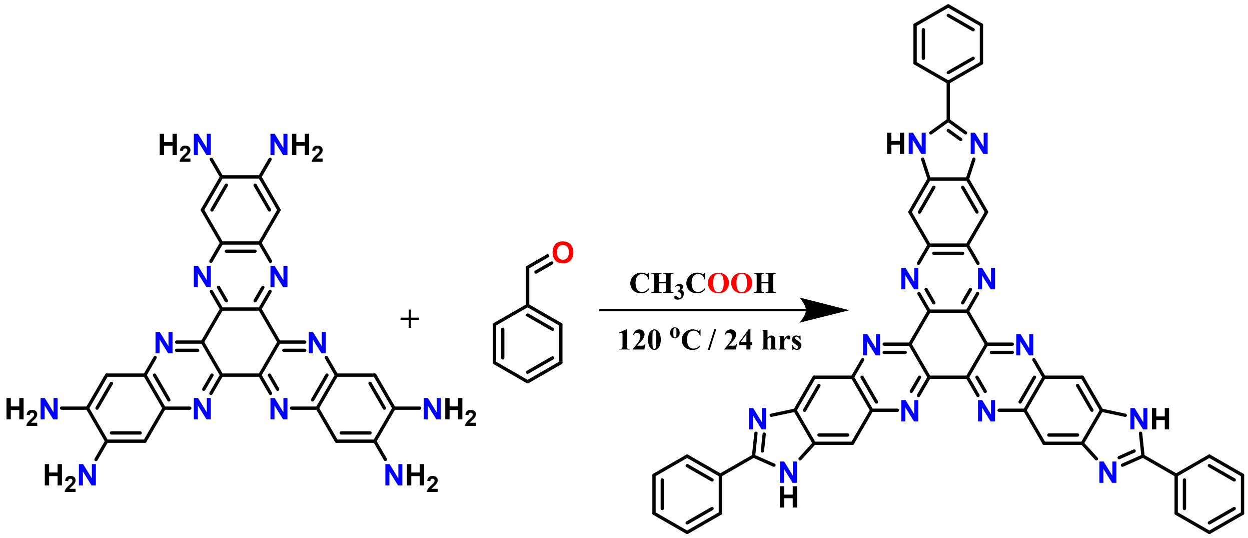

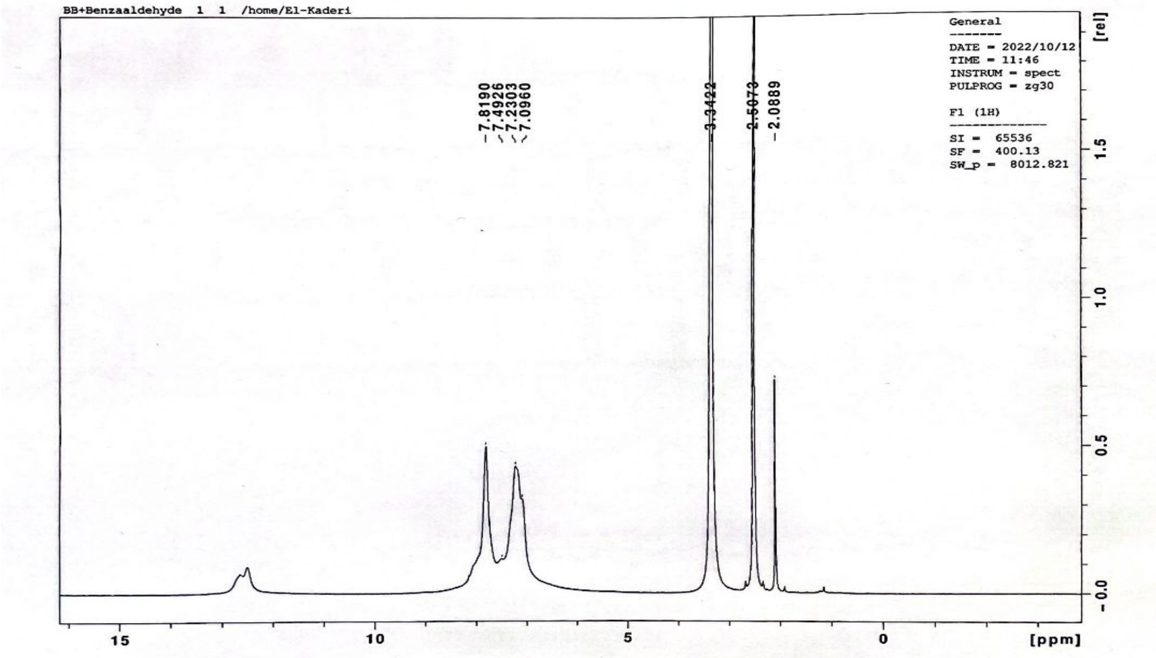

Figure S16. ^{1}HNMR of BCOF-1 monomer. The peaks at 2.088 and 3.342 ppm refer to ethanol and water, respectively.

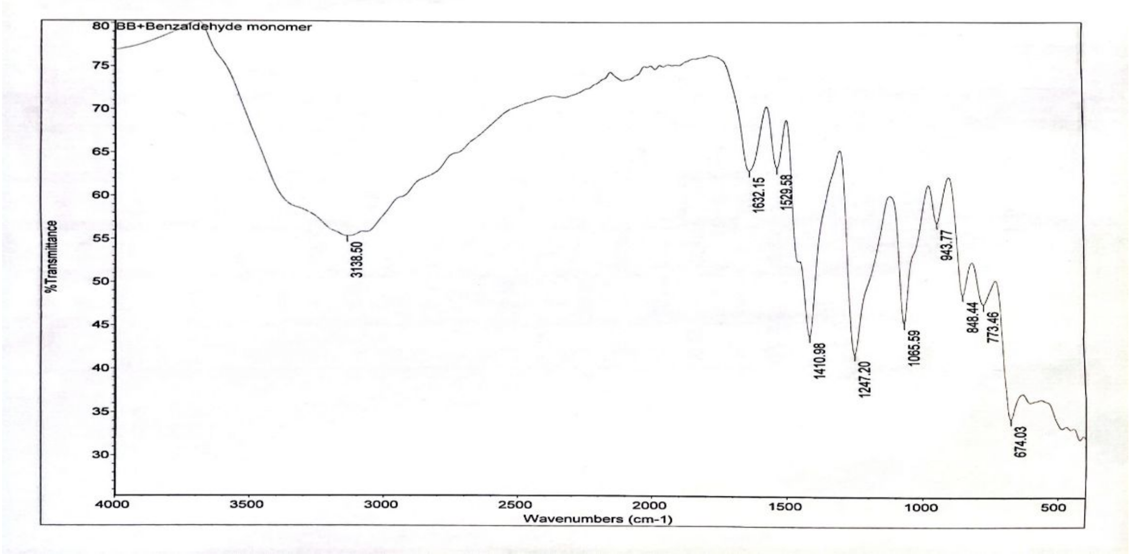

Figure S17. ATR-IR of BCOF-1 monomer.

Synthesis of BCOF-1

A dried Pyrex tube was charged with HATNHA (25.0 mg, 0.0526 mmol) and Terephthalaldehyde (10.5 mg, 0.0783 mmol, 1.5 eq.), 1,4-dioxane (2.0 mL) and mesitylene (0.5 ml), were charged in sequence. The mixture was sonicated for 3 minutes, and the tube was flash-frozen at 77 K using a liquid N_2 bath. After the first freezing, 0.5 ml of 3M CH_3COOH was added then followed by three freeze-pump-thaw cycles. Eventually, the tube was flame-sealed under a vacuum and placed in the oven at 120 °C for 5 days, a dark brown precipitate formed at the bottom of the tube, which was isolated by filtration and washed with DMF (10 ml × 2). The solid was soaked in DMF (20 mL) at room temperature for 2 days, during which the solvent was replaced two times per day. After two days, DMF was replaced with CH_2Cl_2 and kept exchanged with the same solvent 2 times per day for 2 days. Finally, the product was soaked in absolute ethanol and activated using supercritical CO_2 to afford a reddish-brown fluffy precipitate. ATR-IR; 3200 (-N-H), 1610 cm^{-1} (C=N imidazole ring), 1416 cm^{-1} (C-C stretching for the new benzene linker), 1461 and 1241 cm^{-1} for (C=C), 1241 cm^{-1} (C=N) in the pyrazine ring respectively. ^{13}C SS-NMR 156, 144, 137, 128, 110 ppm.

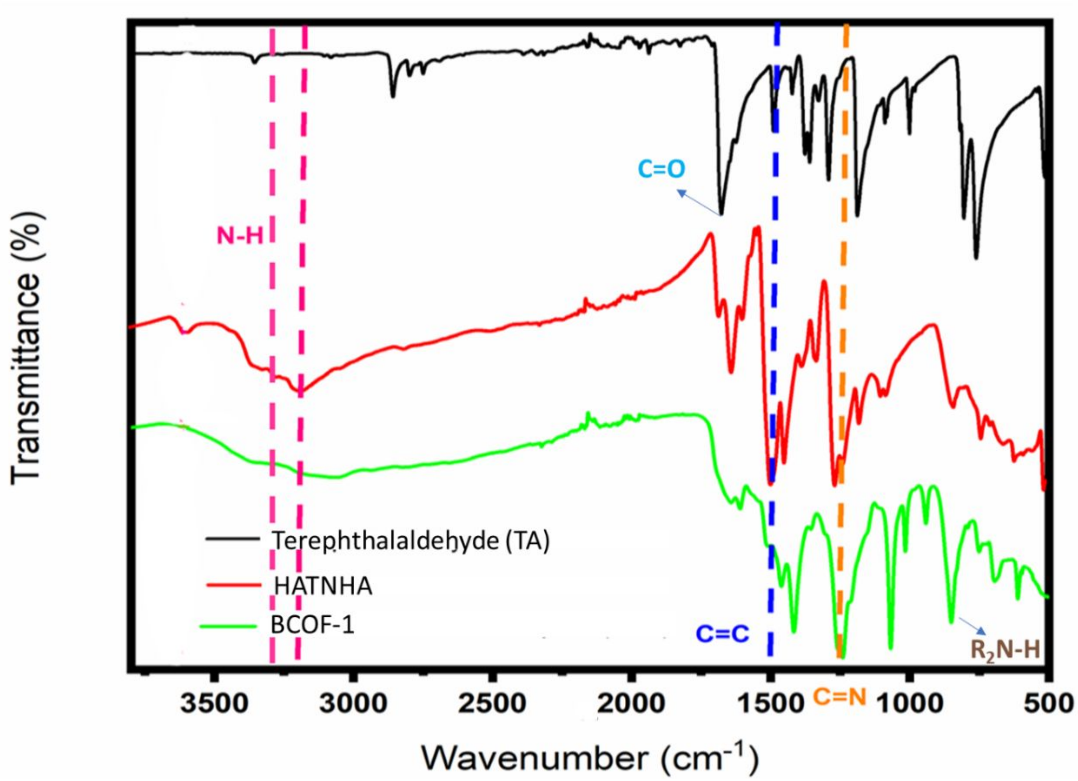

Figure S18. ATR-IR comparison of the precursors and the BCOF-1.

Table S1. Crystallographic parameters for AA stacking BCOF-1.

**Crystallographic parameters for BCOF-1 AA stacking (Eclipsed)**	
Crystal type	Hexagonal
Space group	P6/mm
Source wavelength (Å)	1.5406
a (Å)	37.715697
b (Å)	37.715697
c (Å)	3.44239
α (°)	90
β (°)	90
γ (°)	120
R_p (%)	2.53
R_{wp} (%)	3.31
Refinement method	Pawley

Table S2. Crystallographic parameters of AB stacking BCOF-1.

**Crystallographic parameters for BCOF-1 AB stacking (Staggered)**	
Crystal type	Hexagonal
Space group	$P6_3$/mmc (194)
Source wavelength (Å)	1.5406
a (Å)	37.882282
b (Å)	37.882282
c (Å)	6.360726
α (°)	90
β (°)	90
γ (°)	120

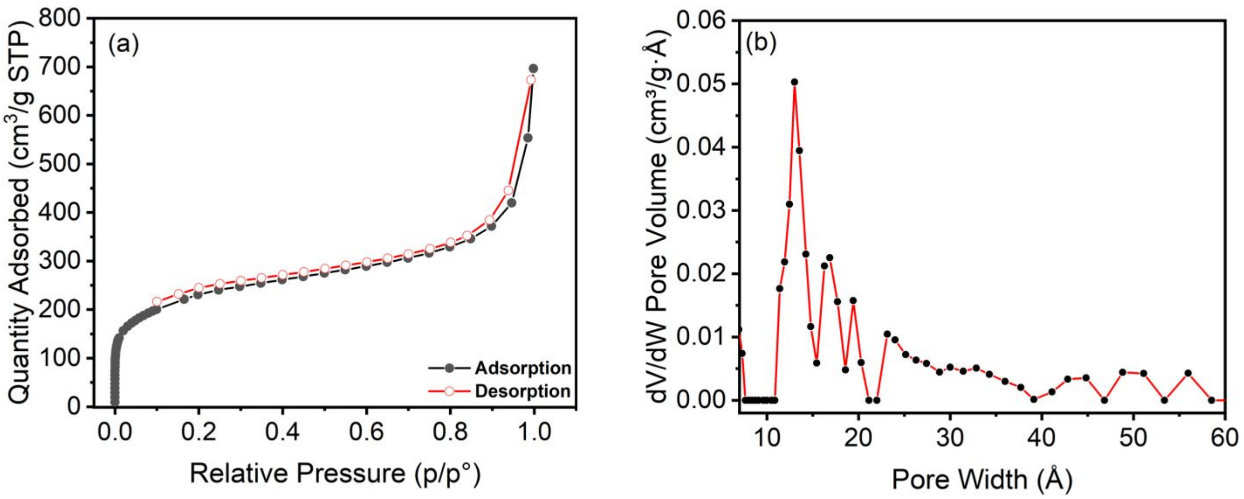

Figure S19. (a) is the N_2 adsorption / desorption isotherm and (b) is the pore size distribution of BCOF-1 showing both microporous up to 20 Å as well as mesopores structures from 20 up to 50 Å.

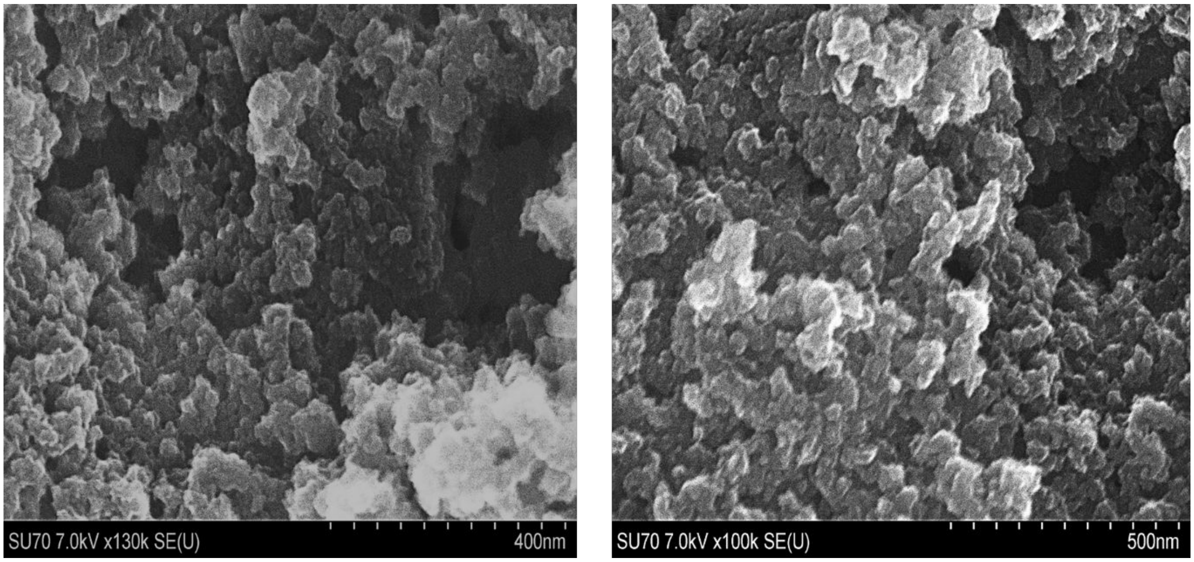

Figure S20. SEM images of as-prepared BCOF-1.

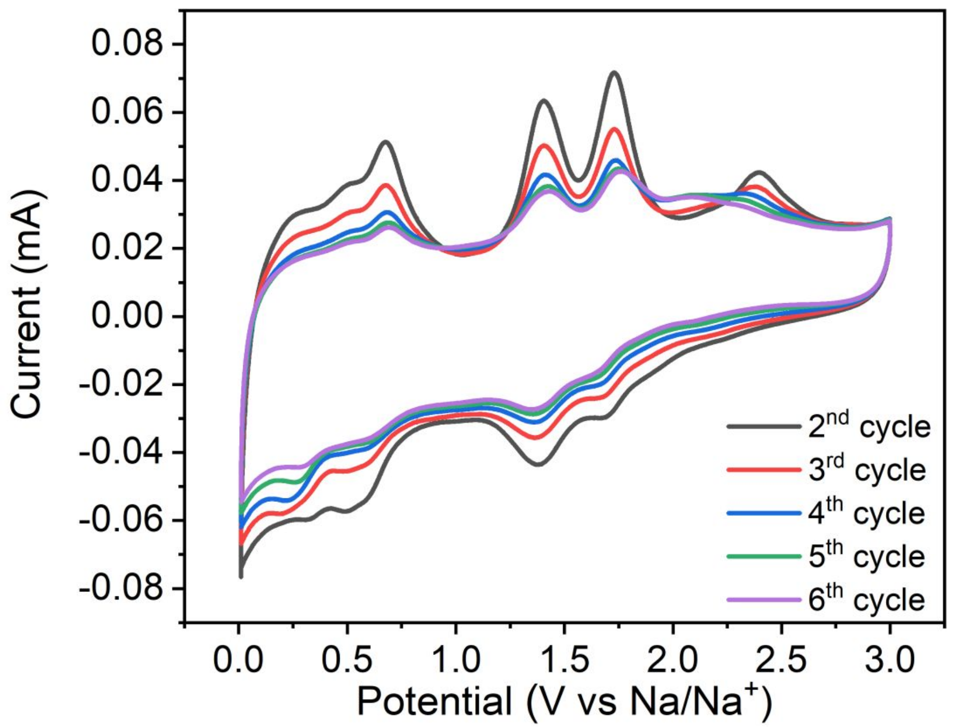

Figure S21. Cyclic voltammograms of BCOF-1 monomer electrodes in 1M $NaPF_6$/ DEGDME at a scan rate of 0.1 mV s^{-1} of the potential range between 0.01 and 3.0 V.

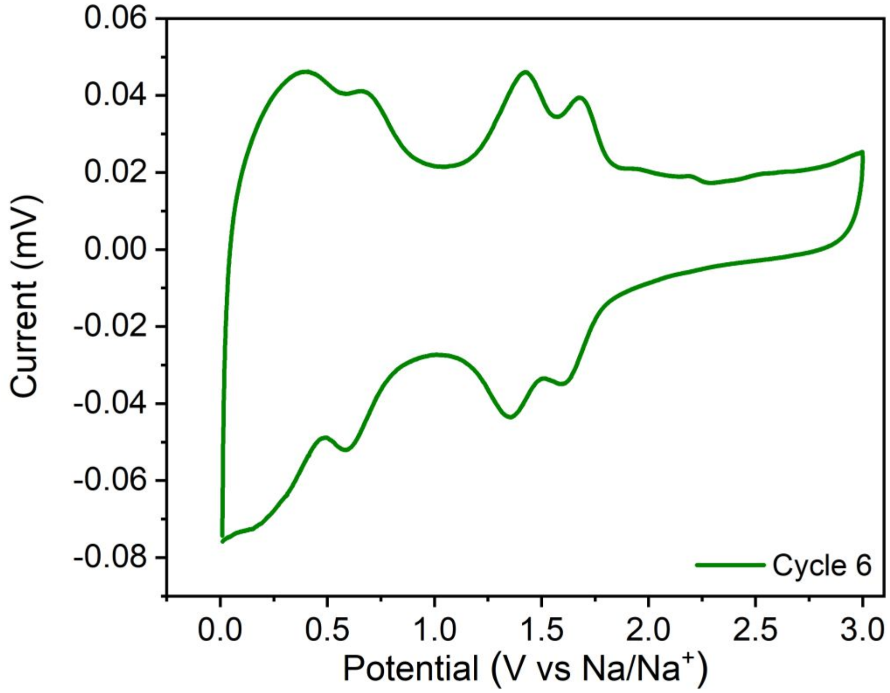

Figure S22. Cyclic voltammograms of BCOF-1 electrodes for just cycle 6 showing the disappearance of the anodic peak at 2.6 upon cycling in 1M $NaPF_6$/ DEGDME at a scan rate of 0.1 mV s^{-1} of the potential range between 0.01 and 3.0 V.

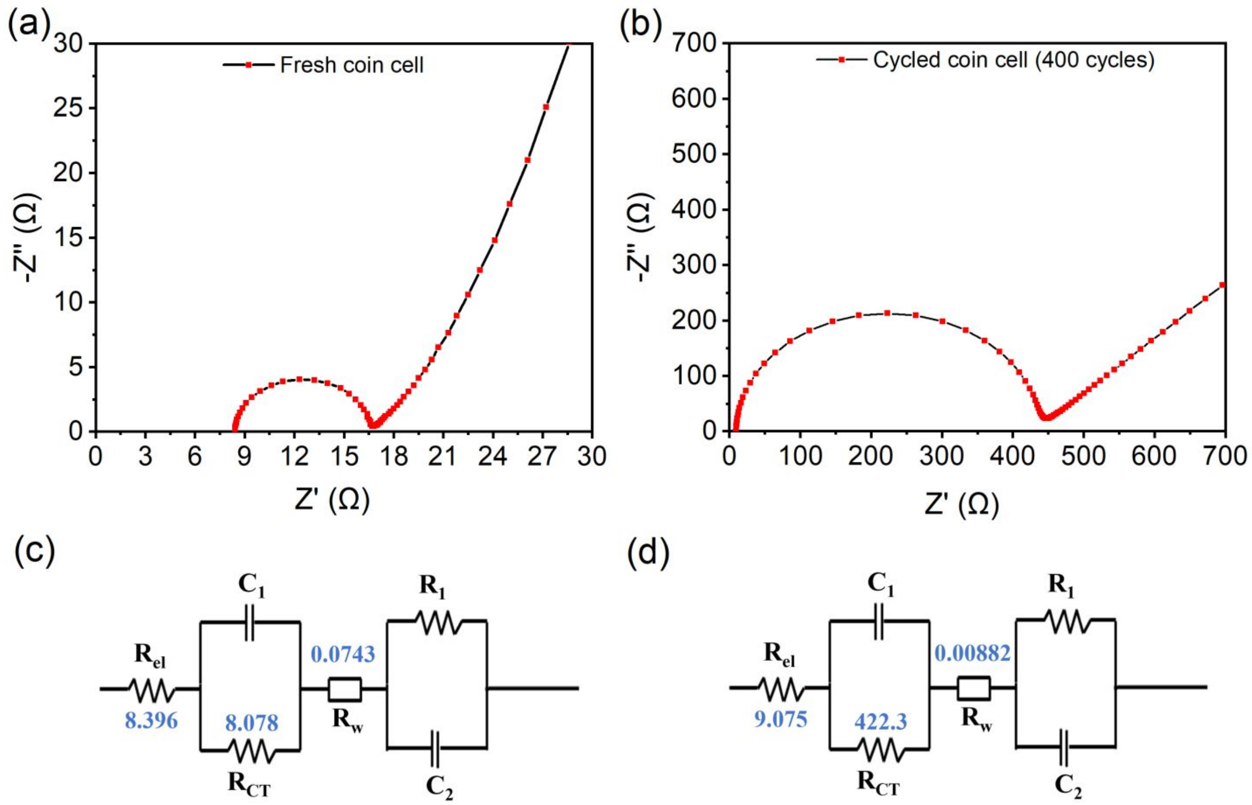

Figure S23. Electrochemical impedance spectra for a) battery before cycling and b) after 400 cycles, and their corresponding equivalent Randles circuit diagrams (c and d).

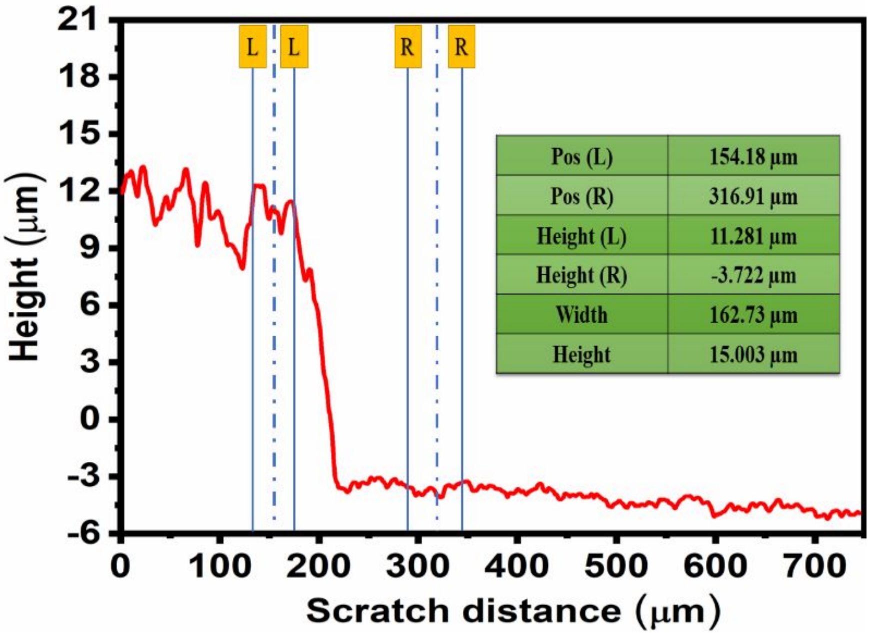

Figure S24. BCOF-1 electrode film thickness measurement (15 µm).

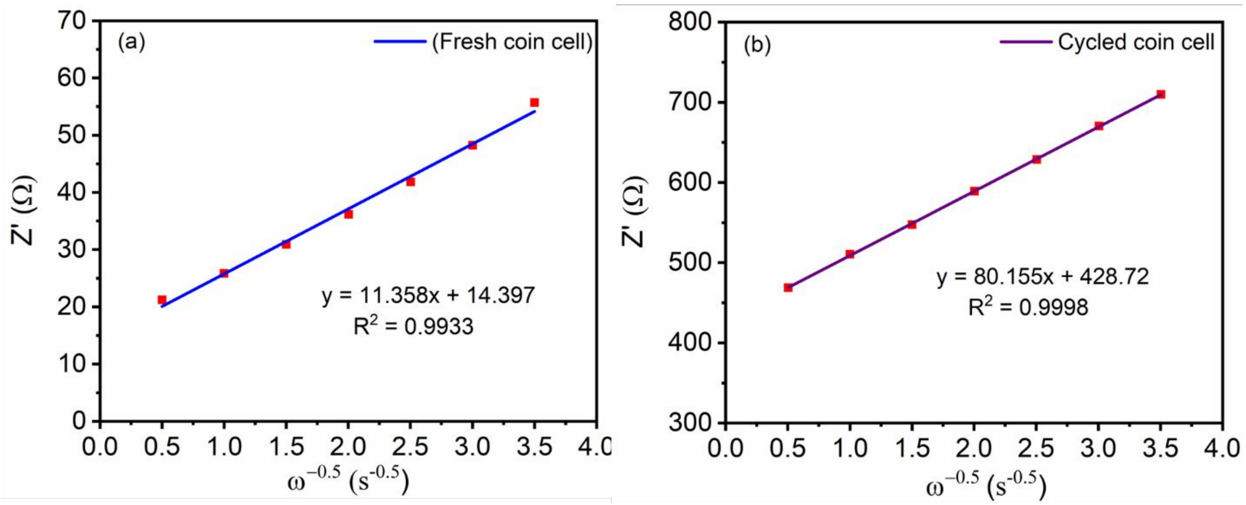

Figure S25. The plot of the real impedance resistance, *Z*′, vs. the reciprocal root square of the lower angular frequencies ($\omega^{-0.5}$) for (a) fresh battery and (b) after being cycled for 400 cycles.

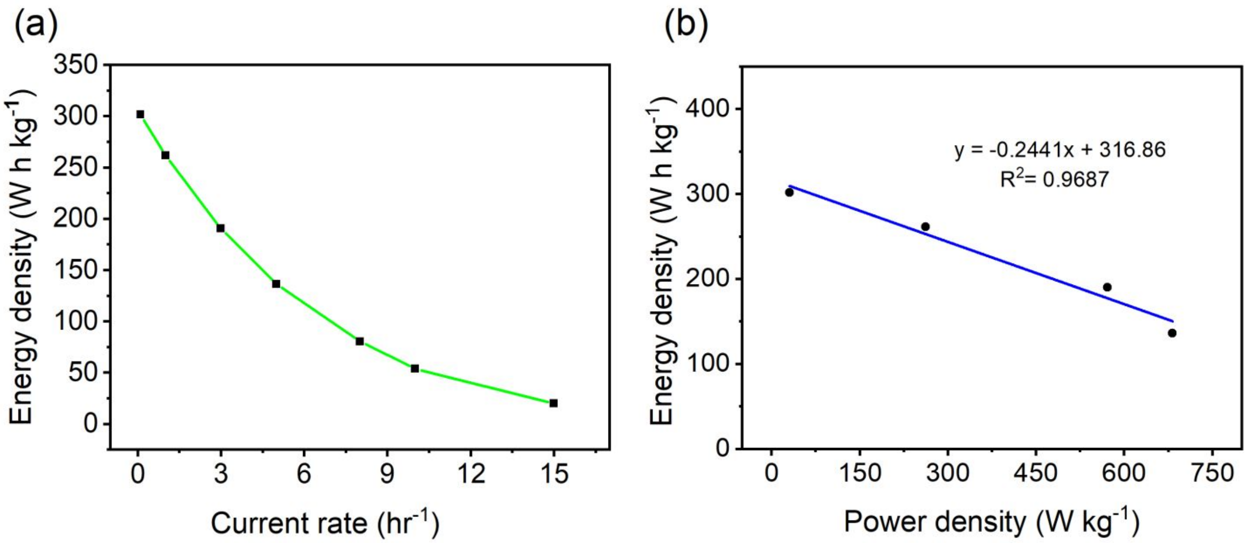

Figure S26. (a) Energy density of BCOF-1based electrode at different current rates of 0.1, 1, 3, 5, 8, 10, and 15 (b) Ragone plot for the 0.1, 1.0, 3.0, and 5.0 C.

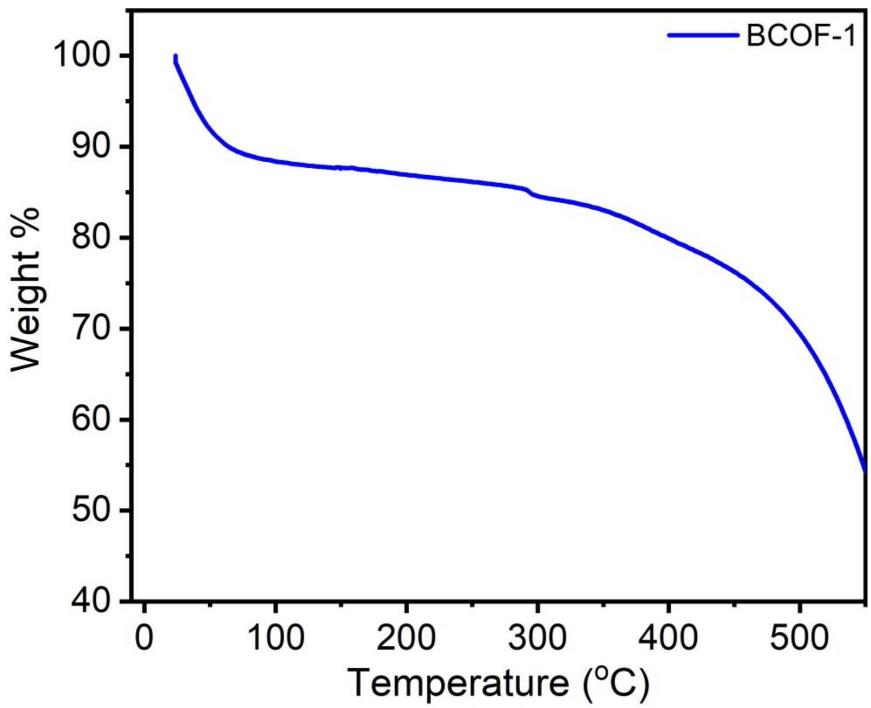

Figure S27. Thermal gravimetric analysis of BCOF-1.

Diffusion coefficient calculations

$$D = \frac{(RT)^2}{2A^2n^2F^4C^2\sigma^2}$$

D is ion diffusion coefficient, R is the molar gas constant (8.314 J mol^{-1} K^{-1}), T is the absolute temperature (298.5 K), A is electrode area (1.767 cm^2), n is the number of electrons transferred in the half-reaction for the redox couple (9), and F is Faraday constant (96484 C mol^{-1}), C is the concentration of sodium ion (mol cm^{-3}), and σ is Warburg coefficient which can be obtained from the following Eq.

$$Z' = R_\Omega + R_{ct} + \sigma\omega^{-1/2}$$

Where σ is the slope for the plot of the real impedance resistance, Z', vs. the reciprocal root square of the lower angular frequencies ($\omega^{-1/2}$). The obtained σ for BCOF-1 before and after 225 cycles are 11.358 and 80.155 Ω $s^{-1/2}$, respectively (Figure S21). Therefore, the diffusion of the calculated coefficients for Na^+ in the BCOF-1were calculated before cycling to be (1.09 x 10^{-12} cm^2 s^{-1}) and after being cycled (2.19 x 10^{-14} cm^2 s^{-1}), respectively.

Energy density and power density calculations

Energy density can be calculated at a certain current rate according to the following equation:

$$Energy\ Density = Discharge\ Potential\ Average * Discharge\ Specific\ Capacity$$

Note: the discharge potential average is not the average of the potential window being used but the discharge potential average after the battery is completely discharged at a certain C rate.

For Power density at a certain current rate is as follow:

$$Power\ Density = Energy\ Density * C\ rate$$

Table S3. Comparison of the sodium-storage performance of BCOF-1with representative covalent organic frameworks SIB electrodes. (AM = active material, PVDF = Polyvinylidene fluoride)

Organic Material	Active site(s)	Sp. Capacity (mA h g^{-1})	Capacity retention	Potential window (V)	AM : C : binder (binder)	Surface area ($m^2 g^{-1}$)	Ref
Aza-COF	-C = N	581 / 0.1 C	87% / 500 0.5 C	0.01-3.0	60:30:10 (Sodium alginate)	240	6
IISERP-COF18	-C = O, -C = N	410 / 0.01 Ag^{-1}	92% /1400 1 Ag^{-1}	0.05-3.0	65:25:10 (PVDF)	1745	7
TQBQ-COF	-C = O, -C = N	452 / 0.02 Ag^{-1}	96% /1000 10 Ag^{-1}	1.0-3.6	50:40:10 (PVDF)	46	8
PHATN	-C=N	220/ 0.125 C	89.2% / 10000 2 Ag^{-1}	1.0-3.5	60:30:10 (PVDF)	-	9
ALP-8	N=N	194/ 0.3 C	90% / 150 0.3 C	0.01-3.0	60:30:10 (Sodium alginate)	550	10
DAPT-TFP-CPF	-C=O	179/ 0.1 Ag^{-1}	92% / 1000 0.1 Ag^{-1}	0.8-3.2	50:40:10 (PVDF)	68	11
DAAQ-COF	-C = O, -C = N	420 / 0.1 Ag^{-1}	99% / 10000 (5 Ag^{-1})	0.05-3.0	60:30:10 (PVDF)	-	12
TFPB-TAPT	-C=N	235/ 0.03 Ag^{-1}	51% / 500 0.03 Ag^{-1}	0.01-1.6	65:25:10	120	13
PID	-C=O	100/0.015 Ag^{-1}	80% /120 0.015 Ag^{-1}	1.5-3.5	80:10:10 (PVDF)	1430	14
DAAQ–HCCP COF	-C=O, -C=N	138 /0.1 Ag^{-1}	88.9% / 1000 2 Ag^{-1}	0.1-2.0	60:30:10 (PVDF)	33	15
NA-NiPc	-C=N	430/ 0.05 Ag^{-1}	127% / 1000 0.2 Ag^{-1}	0.01-3.0	60:30:10 (PVDF)	382	16
PPDA-NiPc	-C=N	385/ 0.05 Ag^{-1}	147% / 1000 0.2 Ag^{-1}	0.01-3.0	60:30:10 (PVDF)	471	16

DAB-NiPc	-C=N	338/ 0.05 Ag^{-1}	139% / 1000<br>0.2 Ag^{-1}	0.01-3.0	60:30:10<br>(PVDF)	575	[16]
E-FCTF	-C=C, -C=N	332/ 0.1 Ag^{-1}	66%/ 200<br>0.1 Ag^{-1}	0.01-2.7	70:20:10<br>(PVDF)	583	[17]
CON-16	-C=N	320/ 0.1 Ag^{-1}	78% / 30<br>0.1 Ag^{-1}	0.01-2.5	70:20:10<br>(Polyacrylic acid)	300	[18]
PICOF-1	-C=O	230/ 0.1 C	84% / 150<br>0.3 C	0.01-3.0	60:30:10<br>(PVDF)	924	[19]
BCOF-1	-C=N	287/0.1 C	77%/400<br>3.0 C	0.01-3.0	50:30:20<br>(Sodium alginate)	840	This Work

References

(1) Shang, X.; Li, X.; Xi, N.; Zhai, Y.; Zhang, J.; Xu, X. Theory and Experiment: Recognition Properties of Chemosensor Containing Ruthenium(II) System in Water Solution. *Sensors Actuators, B Chem.* **2011**, *160* (1), 1112–1119.

(2) Shang, X. F.; Lin, H.; Lin, H. K. The Synthesis and Recognition Properties of Colorimetric Fluoride Receptors Bearing Sulfonamide. *J. Fluor. Chem.* **2007**, *128* (5), 530–534.

(3) Kleineweischede, A.; Mattay, J. Synthesis of Amino- and Bis(Bromomethyl)-Substitued Bi- and Tetradentate N-Heteroaromatic Ligands: Building Blocks for Pyrazino-Functionalized Fullerene Dyads. *European J. Org. Chem.* **2006**, No. 4, 947–957.

(4) Skujins, S.; Webb, G. A. Spectroscopic and Structural Studies of Some Oxocarbon Condensation Products—III. *Tetrahedron* **1969**, *25* (17), 3955–3960.

(5) Kim, S. Synthesis of Dimensional Organic Networks as Energy Materials. *Graduate School of UNIST*, **2019**, 1–112.

(6) Shehab, M. K.; Weeraratne, K. S.; Huang, T.; Lao, K. U.; El-Kaderi, H. M. Exceptional Sodium-Ion Storage by an Aza-Covalent Organic Framework for High Energy and Power Density Sodium-Ion Batteries. *ACS Appl. Mater. Interfaces* **2021**, *13* (13), 15083–15091.

(7) Haldar, S.; Kaleeswaran, D.; Rase, D.; Roy, K.; Ogale, S.; Vaidhyanathan, R. Tuning the Electronic Energy Level of Covalent Organic Frameworks for Crafting High-Rate Na-Ion Battery Anode. *Nanoscale Horizons* **2020**, *5* (8), 1264–1273.

(8) Shi, R.; Liu, L.; Lu, Y.; Wang, C.; Li, Y.; Li, L.; Yan, Z.; Chen, J. Nitrogen-Rich Covalent Organic Frameworks with Multiple Carbonyls for High-Performance Sodium Batteries. *Nat. Commun.* **2020**, *11* (1), 178.

(9) Mao, M.; Luo, C.; Pollard, T. P.; Hou, S.; Gao, T.; Fan, X.; Cui, C.; Yue, J.; Tong, Y.; Yang, G.; Deng, T.; Zhang, M.; Ma, J.; Suo, L.; Borodin, O.; Wang, C. A Pyrazine-Based Polymer for Fast-Charge Batteries. *Angew. Chemie - Int. Ed.* **2019**, *58* (49), 17820–17826.

(10) Weeraratne, K. S.; Alzharani, A. A.; El-Kaderi, H. M. Redox-Active Porous Organic Polymers as Novel Electrode Materials for Green Rechargeable Sodium-Ion Batteries. *ACS Appl. Mater. Interfaces* **2019**, *11* (26), 23520–23526.

(11) Li, H.; Tang, M.; Wu, Y.; Chen, Y.; Zhu, S.; Wang, B.; Jiang, C.; Wang, E.; Wang, C. Large π-Conjugated Porous Frameworks as Cathodes for Sodium-Ion Batteries. *J. Phys. Chem. Lett.* **2018**, *9* (12), 3205–3211.

(12) Gu, S.; Wu, S.; Cao, L.; Li, M.; Qin, N.; Zhu, J.; Wang, Z.; Li, Y.; Li, Z.; Chen, J.; Lu, Z. Tunable Redox Chemistry and Stability of Radical Intermediates in 2D Covalent Organic Frameworks for High Performance Sodium Ion Batteries. *J. Am. Chem. Soc.* **2019**, *141* (24), 9623–9628.

(13) Patra, B. C.; Das, S. K.; Ghosh, A.; Raj, A. K.; Moitra, P.; Addicoat, M.; Mitra, S.; Bhaumik, A.; Bhattacharya, S.; Pradhan, A. Covalent Organic Framework Based Microspheres as an Anode Material for Rechargeable Sodium Batteries. *J. Mater. Chem.*

A **2018**, *6* (34), 16655–16663.

(14) Van Der Jagt, R.; Vasileiadis, A.; Veldhuizen, H.; Shao, P.; Feng, X.; Ganapathy, S.; Habisreutinger, N. C.; Van Der Veen, M. A.; Wang, C.; Wagemaker, M.; Van Der Zwaag, S.; Nagai, A. Synthesis and Structure-Property Relationships of Polyimide Covalent Organic Frameworks for Carbon Dioxide Capture and (Aqueous) Sodium-Ion Batteries. *Chem. Mater.* **2021**, *33* (3), 818–833.

(15) Hu, M.; Huang, H.; Gao, Q.; Tang, Y.; Luo, Y; Deng, Y.; Zhang, L. Anthraquinone-Based Covalent Organic Framework Nanosheets with Ordered Porous Structures for Highly Reversible Sodium Storage. *Energy & Fuels* **2021**, *35*, 1851–1858.

(16) Zhao, J.; Zhou, M.; Chen, J.; Tao, L.; Zhang, Q.; Li, Z.; Zhong, S.; Fu, H.; Wang, H.; Wu, L. Phthalocyanine-Based Covalent Organic Frameworks as Novel Anode Materials for High-Performance Lithium-Ion/Sodium-Ion Batteries. *Chem. Eng. J.* **2021**, *425*, 131630.

(17) Zhang, H.; Sun, W.; Chen, X.; Wang, Y. Few-Layered Fluorinated Triazine-Based Covalent Organic Nanosheets for High-Performance Alkali Organic Batteries. *ACS Nano* **2019**, *13* (12), 14252–14261.

(18) Kim, M. S.; Lee, W. J.; Paek, S. M.; Park, J. K. Covalent Organic Nanosheets as Effective Sodium-Ion Storage Materials. *ACS Appl. Mater. Interfaces* **2018**, *10* (38), 32102–32111.

(19) Shehab, M. K.; Weeraratne, K. S.; El-Kadri, O. M.; Yadavalli, V. K.; El-Kaderi, H. M. Templated Synthesis of 2D Polyimide Covalent Organic Framework for Rechargeable Sodium-Ion Batteries. *Macromol. Rapid Commun.* **2022**, 2200782.
